# Distinct 2-phenylimidazo[1,2-*a*]pyridine derivatives that inhibit breast cancer cell proliferation identified as AHR ligands

**DOI:** 10.1016/j.isci.2026.115936

**Published:** 2026-04-29

**Authors:** Katrin Koellisch, Christine Blattner, Stefano Motta, Janine Wesslowski, Melanie Rothley, Simone Büchel, Savannah Sirounian, Ilenia Segatto, Hanna T. Weber, Julia Müller, Marina Grimaldi, Jutta Stober, Zoe Wammetsberger, Mengwu Pan, René Houtman, Christoph W. Grathwol, Lo-Wei Lin, Laki Buluwela, Siva Kumar Kolluri, Dominik Mytzka, Simak Ali, Nicole Jung, Patrick Balaguer, Sonja Thaler, Barbara Belletti, Laura Bonati, William Bourguet, Stefan Bräse, Gary Davidson, Andrew C.B. Cato

**Affiliations:** 1 Institute of Biological and Chemical Systems - Functional Molecular Systems, Karlsruhe Institute of Technology, Kaiserstraße 12, 76131 Karlsruhe, Germany; 2 Institute of Biological and Chemical Systems - Biological Information Processing, Karlsruhe Institute of Technology, Kaiserstraße 12, 76131 Karlsruhe, Germany; 3 Department of Earth and Environmental Sciences, University of Milano-Bicocca, Piazza della Scienza 1, 20126 Milan, Italy; 4 CBS (Centre de Biologie Structurale), University Montpellier, CNRS, Inserm, Montpellier, France; 5 Unit of Molecular Oncology, Centro di Riferimento Oncologico di Aviano (CRO), IRCCS, National Cancer Institute, Aviano, Italy; 6 European Center for Angioscience, Medical Faculty Mannheim, University of Heidelberg, 68167 Mannheim, Germany; 7 IRCM (Institut de Recherche en Cancérologie de Montpellier), Inserm U1194, University Montpellier, ICM, Montpellier, France; 8 Precision Medicine Lab, Oss, the Netherlands; 9 Cancer Research Laboratory, Department of Environmental and Molecular Toxicology, Oregon State University, Corvallis, OR, USA; 10 Department of Surgery and Cancer, Imperial College London, London W12 0NN, UK; 11 Linus Pauling Institute, Oregon State University, Corvallis, OR, USA; 12 Institute of Pharmacy and Molecular Biotechnology IPMB, Heidelberg University, 69120 Heidelberg, Germany; 13 Institute of Organic Chemistry, Karlsruhe Institute of Technology, Kaiserstraße 12, 76131 Karlsruhe, Germany

**Keywords:** cell biology, cancer

## Abstract

X15695 is a 2-phenylimidazo[1,2-*a*] pyridine derivative previously described as an orally active, selective estrogen receptor (ER) degrader that inhibits the proliferation of ER^+^ breast cancer cells. Here, we show that X15695 and derivatives are aryl hydrocarbon receptor (AHR) ligands. Knockout of AHR abolishes the anti-proliferative property of the imidazopyridine derivatives. In the presence of estradiol, X15695 and derivatives outperform the standard of care drug fulvestrant in suppressing the growth of ER^+^ breast cancer cells, expressing either the wild-type or clinically relevant ER mutant forms (Y537S and D538G) and of patient-derived organoids established from ER^+^ tumors. Using computational techniques, we discovered that a low pKa value resulting from electron-withdrawing substituents in the 2-phenylimidazo[1,2-*a*] pyridine compounds is a key feature that identifies them as potent AHR ligands, leading to the potential discovery of additional derivatives for future therapeutic development.

## Highlights

• 2-phenylimidazopyridines inhibit wildtype and mutant ER+ breast cancer cell growth
• They are orally bioavailable and induce ERα degradation but lack direct ER binding
• They outperform fulvestrant in preclinical breast cancer models (CTCs, PDO, PDxO)
• These compounds have now been identified as *bona fide* AHR ligands

## Graphical abstract

**Figure:**
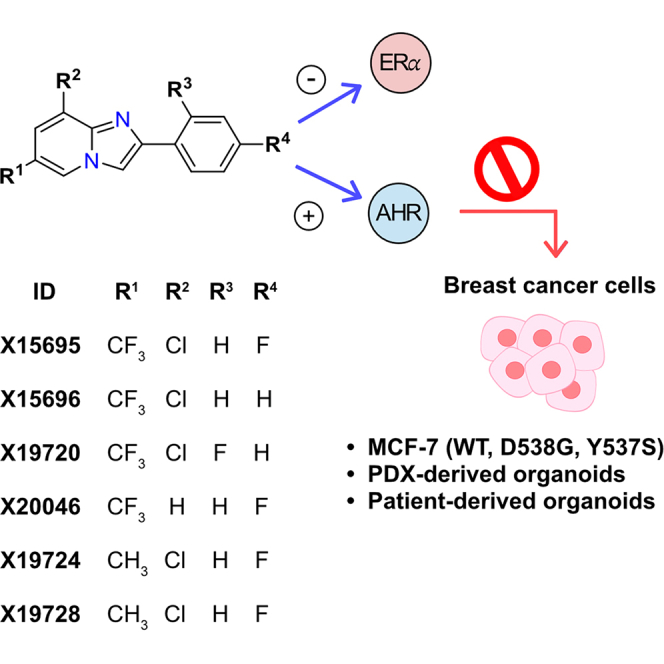

## Introduction

Breast cancer is one of the most frequently diagnosed cancers and a leading cause of cancer death in women worldwide.^1^ About 70% of all breast cancers rely on hormone signaling for growth and express the estrogen and/or progesterone hormone receptors (ER, PR), but are negative for the human epidermal growth factor receptor 2 (HER2).^2^ Current therapies targeting the hormone receptors include inhibitors of estrogen production, such as luteinizing hormone-releasing hormone (LHRH) agonists, aromatase inhibitors (AIs), or selective estrogen receptor modulators (SERMs) such as tamoxifen and selective estrogen receptor degraders (SERDs) such as fulvestrant.^3^

These endocrine therapies have significantly improved the outcome of hormone receptor-positive breast cancer, but there are shortcomings resulting from *de novo* and acquired endocrine resistance.^4^ Acquired resistance frequently arises from increased mutations in the ligand-binding domain of the estrogen receptor alpha gene (*ESR1*).^5^ Such mutations are uncommon in primary or treatment-naïve primary tumors but occur frequently during therapies that target estrogen signaling pathways.^6^^,^^7^ The *ESR1* mutations promote estrogen-independent constitutive activation of ER and estrogen-independent growth^8^ and are detected in nearly 30% of all patients with ER^+^ metastatic disease. Of these, more than half are accounted for by the variant Y537S (21%) and D538G (33%).^9^ The Y537S mutation, in particular, is associated with a higher degree of resistance to most endocrine therapies.^8^^,^^9^^,^^10^^,^^11^

The first clinically approved SERD, fulvestrant, showed only modest inhibition of mutant ERα, compared to wild-type ERα.^3^ Fulvestrant also has some limitations in its clinical use due to its intramuscular formulation and once-a-month injection.^12^ New inhibitors with superior bioavailability and ER-degrading potential (oral SERDs) have therefore been developed,^12^^,^^13^ and these include elacestrant, giredestrant, amcenestrant, camizestrant, and imlunestrant.^12^^,^^13^^,^^14^ Although additional SERDs with improved mutant ERα inhibition are still in development, some studies suggest that the degree of degradation of ERα by SERDs may not be sufficient for clinical benefit in tumors expressing only wild-type ER, especially when they are used as monotherapy.^15^^,^^16^ It is therefore important to identify and develop other compounds that have a mechanism of action different from SERDs.

We have recently described 2-phenylimidazo[1,2-*a*] pyridine derivatives as potent inhibitors of breast and prostate cancer cell growth in preclinical studies. The prototype compound X15695 was shown to degrade ERα and inhibit the growth of MCF-7 breast cancer xenografts when administered orally, and it was therefore classified as an oral SERD.^17^ In addition to ERα degradation, X15695 activated p53 and induced cell cycle block and apoptosis, however, its primary target remained unidentified. In this study, we have shown that X15695 and further derivatives do not interact with the ERα and can therefore not be described as SERDs. Rather, we have identified them as aryl hydrocarbon receptor (AHR) ligands that enhance the transactivation function of the AHR. In the presence of estradiol, X15695 and derivatives outperformed fulvestrant in inhibiting the proliferation of MCF-7 cells expressing the clinically relevant ER mutations that confer resistance to current endocrine therapies. Importantly, favorable profiles of X15695 were also confirmed in preclinical models, using patient-derived organoids (PDOs) as well as organoids from patient-derived xenograft (PDxO), established from patients with ER^+^ breast cancer. Indeed, efficacy of X15695 has also been demonstrated in circulatory tumor cells (CTCs) isolated from a breast cancer patient with acquired endocrine resistance.^18^^,^^19^ We have therefore identified unique properties of a novel class of compounds that can be additionally developed for breast cancer therapy.

## Results

### Imidazopyridines outperform fulvestrant in inhibiting the proliferation of breast cancer cells

**Figure 1:**
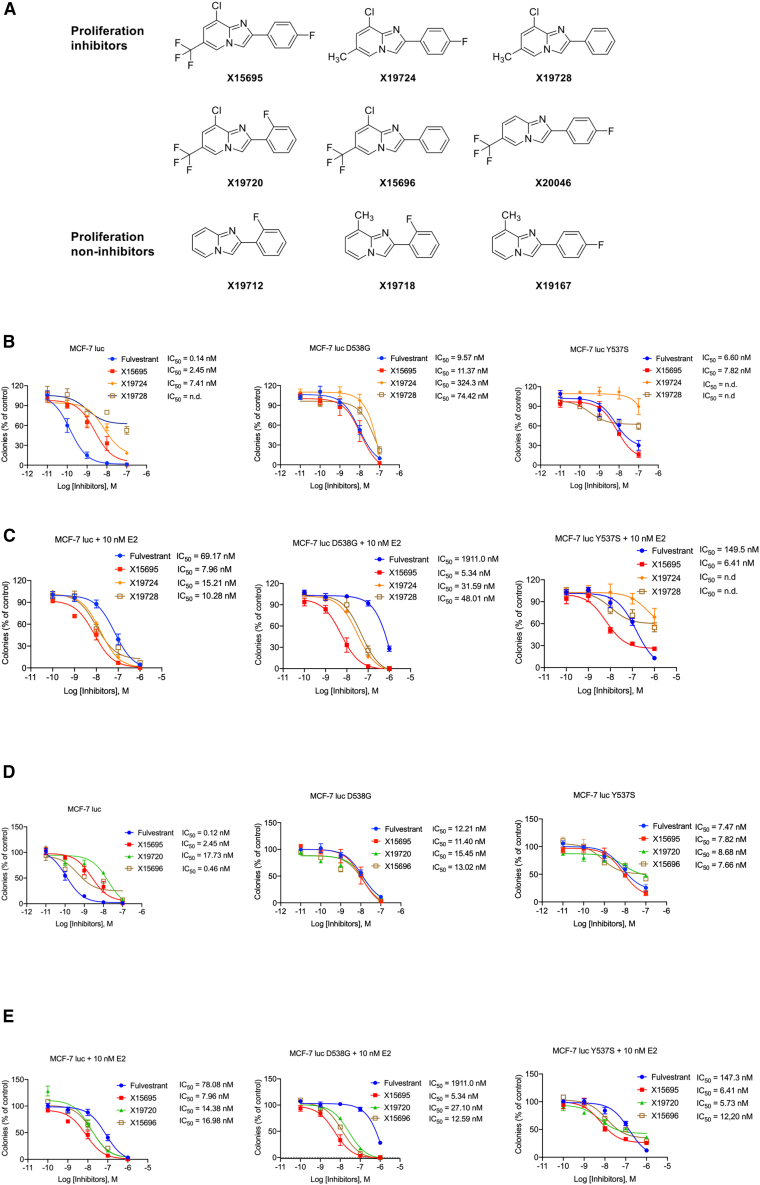
Imidazopyridines outperform fulvestrant in inhibiting the proliferation of breast cancer (A) Structure of 2-phenylimidazo[1,2-*a*] pyridine derivatives used in this study and previously identified as inhibitors and non-inhibitors of ER^+^ breast cancer cell proliferation.^17^ (B–E) Comparison of the action of imidazopyridine derivatives and fulvestrant in the clonal expansion of MCF-7 cells expressing the wild-type ER or the D538G of Y537S ER mutations. Cells were treated with and without the indicated compounds in the presence and absence of E_2_ (10 nM) for 14 days. The colonies were stained and photographed, and the areas covered by the colonies were calculated using the ColonyArea Plugin for Fiji (RRID: SCR_003070).^20^ The results represent the mean ± SEM (*n* = 3–8) in the absence (B and D) or presence (C and E) of 10 nM E_2_.

We previously identified six 2-phenylimidazo[1,2-*a*] pyridine derivatives that inhibited the proliferation of ER^+^ MCF-7, ZR75–1, and T47D but not MDA-MB231 ER^−^ breast cancer cells.^17^ However, the mechanism of action of these compounds remains unclear. We selected three of the six inhibitory compounds (X15695, X19724, and X19728) (Figure 1A) and analyzed their ability to inhibit the clonal expansion of MCF-7 breast cancer cells that express the wild-type or knock-in ERα mutations D538G and Y537S identified in patients with endocrine-resistant breast cancer.^21^ The rationale for this selection was to determine the consequences of replacing the trifluoromethyl group at the C-6 position of X15695 with a methyl group in X19724 or X19728. The trifluoromethyl group is important in medicinal Chemistry for enhancing metabolic stability, lipophilicity, and bioavailability,^22^ and substituents at the C-6 position of the imidazopyridine scaffold are known to critically influence a compound’s biological and pharmacological activity.^23^

In MCF-7 cells expressing the wild-type ERα, the standard of care drug fulvestrant was more potent than X15695 in inhibiting clonal expansion (fulvestrant IC_50_ = 0.14 nM vs. X15695 IC_50_ = 2.45 nM) but was as potent as fulvestrant in cells expressing the two mutant ERs (fulvestrant IC_50_ = 6.60–9.57 nM vs. X15695 IC_50_ = 7.82–11.63 nM) (Figure 1B). X19724 and X19728 were much weaker inhibitors of clonal expansion of both the wild-type and mutant ER-expressing cells, consistent with the reported outcome of replacing a trifluoromethyl group.^22^ As the serum levels of estradiol (E_2_) in patients with premenopausal breast cancer undergoing anti-estrogen therapy can reach nanomolar concentrations (approx. 277.9 pg/mL or 1.02 nM),^24^ we evaluated the inhibition of clonal formation in the presence of E_2_ to achieve a situation of fully liganded ERα. Intriguingly, the inhibitory action of fulvestrant compared to X15695 was significantly reduced in the presence of 10 nM estradiol (E_2_) (Figure 1C), varying in potency from 69.17 nM in cells expressing the wild-type receptor to the micromolar range (0.15 μM–1.91 μM) in cells expressing the mutant receptors. On the other hand, the action of X15695 remained relatively unchanged by E_2_ treatment (IC_50_ = 5–6 nM in the ER mutant cells compared to 7 nM in the WT cells) (Figure 1C), while X19724 and X19728 still remained relatively weak, especially in the mutant ER expressing cells (Figure 1C). To demonstrate that the reduced anti-proliferative effect of X19724 and X19728 was due to the CH3 substitutions, we analyzed two other compounds that carry C-6 CF3 substituents (X19720 and X15696). Note that X15696 is structurally identical to X19728 except for CF3 instead of CH3 at position C-6. As expected, X19720 and X15696 no longer exhibited the reduced anti-proliferative action seen for X19724 and X19728 (Figures 1D and 1E), and, such as X15695, they also outperformed fulvestrant in the proliferation inhibition of MCF-7 cells in the presence of E_2_ (Figure 1E).

**Figure 2:**
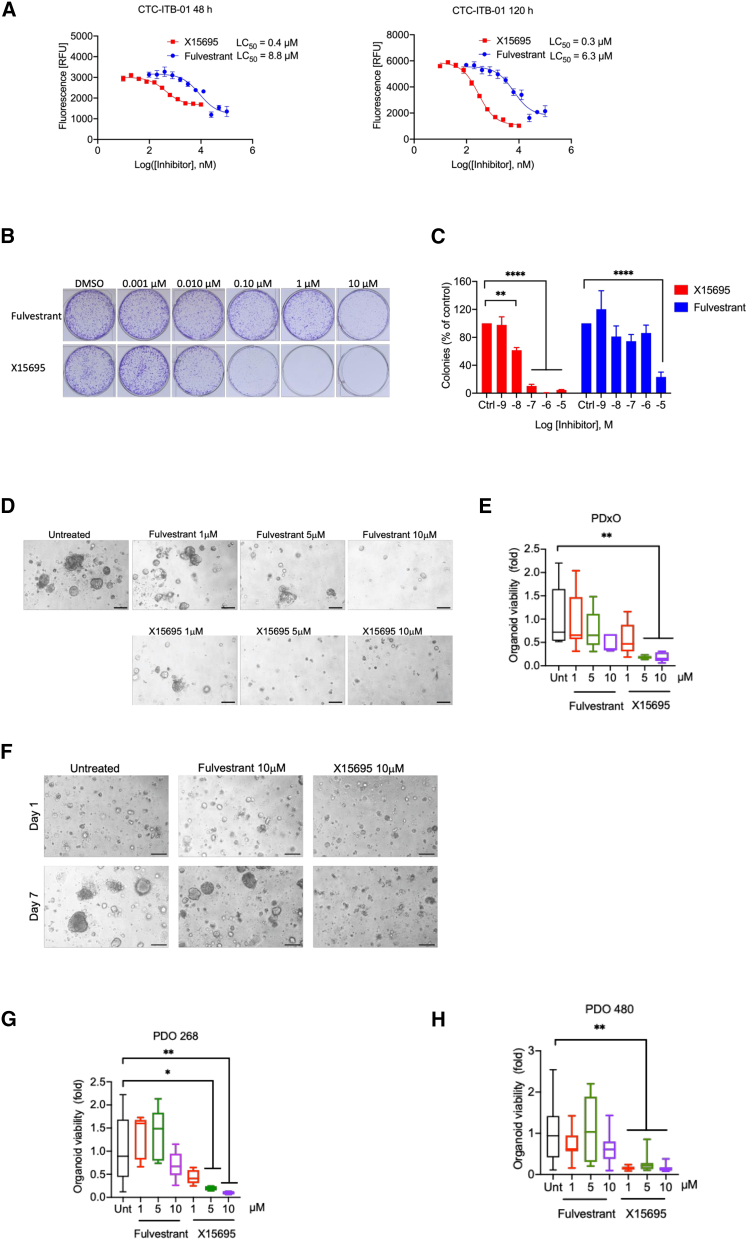
Imidazopyridines outperform fulvestrant in reducing the viability of ER^+^ breast cancer patient-derived models Reduction of proliferation of the ER+ breast cancer cell line derived from the CTC of an endocrine-resistant breast cancer patient. (A) Determination of LC_50_ values for X15695 and fulvestrant in CTC-ITB-01 at 48 and 120 h using CyQUANT kit. Cells were treated with serial dilutions of each inhibitor, and the graphs depict relative fluorescence intensities plotted as a function of the logarithm of the inhibitor concentration (nM). LC_50_ values were calculated with GraphPad Prism with a 95% confidence interval (*n* = 4). (B) Representative images of clonogenic growth of CTC-ITB-01 cells following two weeks of treatment with the indicated concentrations of X15695 and fulvestrant. Colonies were fixed and stained with 0.5% Crystal violet solution. (C) Quantification of colony expansion was performed using the ColonyArea plugin for ImageJ. Growth was measured as the area covered by colonies, normalized to DMSO-treated control wells. Results are shown as bar plots (mean ± SEM, n = 3–4). Statistical significance was assessed by two-way ANOVA (∗∗*p* < 0.01; ∗∗∗∗ <0.0001). DMSO was used as a vehicle control for both compounds. (D) Representative contrast phase images of PDxO#28, treated with vehicle (untreated), fulvestrant, or X15695 at the indicated concentrations. Images were acquired using a 4× objective. Scale bars are 50 μm. (E) Viability of PDxO#28, quantified using Cell Titer Glo reagent. Organoids were embedded and cultured in 3D-Matrigel, then treated with vehicle (Unt), fulvestrant, or X15695 at the indicated doses for 7 days. Data are presented as fold change relative to the untreated condition (n = 5–8). Boxplots show median, minimum, and maximum values of the different experiments. (F) Representative contrast phase images of PDO#268, treated with vehicle (untreated), fulvestrant, or X15695 at the indicated doses for one day or 7 days. Images were acquired using a 4× objective. Scale bars are 50 μm. (G and H) Viability of PDO#268 (G) and PDO#480 (H), quantified using Cell Titer Glo reagent and treated with increasing concentrations of fulvestrant or X15695 for 7 days as described in E. In all boxplots, statistical significance was assessed by one-way ANOVA test, ∗*p* < 0.05; ∗∗< 0.01.

We next compared the ability of X15695 and fulvestrant to overcome acquired endocrine resistance in breast cancer cells that do not harbor *ESR1* mutations. We used CTC-ITB-01, a recently established cell line from circulating tumor cells of a patient with metastatic ER^+^ breast cancer resistant to endocrine therapy,^18^^,^^19^ and evaluated the lethality of the two compounds in a cytotoxicity assay after 48 h and 120 h exposure. At both time points, the lethal dose at which death occurs in 50% of the cells (LC_50_) was 20-fold lower for X15695 compared to fulvestrant (0.4 and 0.3 μM for X15695 compared to 8.8 and 6.3 μM for fulvestrant) (Figure 2A), demonstrating a higher sensitivity of the cells to X15695. Using clonogenic assays, we also showed that X15695 was about three orders of magnitude more potent in inhibiting the clonal expansion of the CTC-ITB-01 cells compared to fulvestrant (Figures 2B and 2C).

To further compare the inhibitory action of X15695 and fulvestrant, we used PDOs and organoids derived from PDxO, established from patients with ER^+^ breast cancer.^25^ PDOs preserve critical features of the original tumors, including genetic and epigenetic profiles, as well as aspects of tissue architecture and cellular heterogeneity making them robust and clinically relevant models for predicting therapeutic responses.^26^

We first used a previously characterized PDxO#28^25^ established from a PDX of a treatment naive ER^+^/HER2^-^ breast cancer patient and embedded them in 3D Matrigel for 24 h followed by a 7 days treatment with increasing doses of fulvestrant or X15695. Viability assays showed that X15695 significantly reduced survival in a dose-dependent manner, whereas the response to fulvestrant was modest (Figures 2D and 2E). The same results were obtained using two additional PDOs, directly established from tumor tissue samples generated from a treatment naive (PDO#268) and a neo-adjuvant pre-treated (PDO#480) ER^+^/HER2^-^ breast cancer patient (Figures 2F–2H).

### Imidazopyridines inhibit breast cancer cell proliferation independent of direct ERα binding

**Figure 3:**
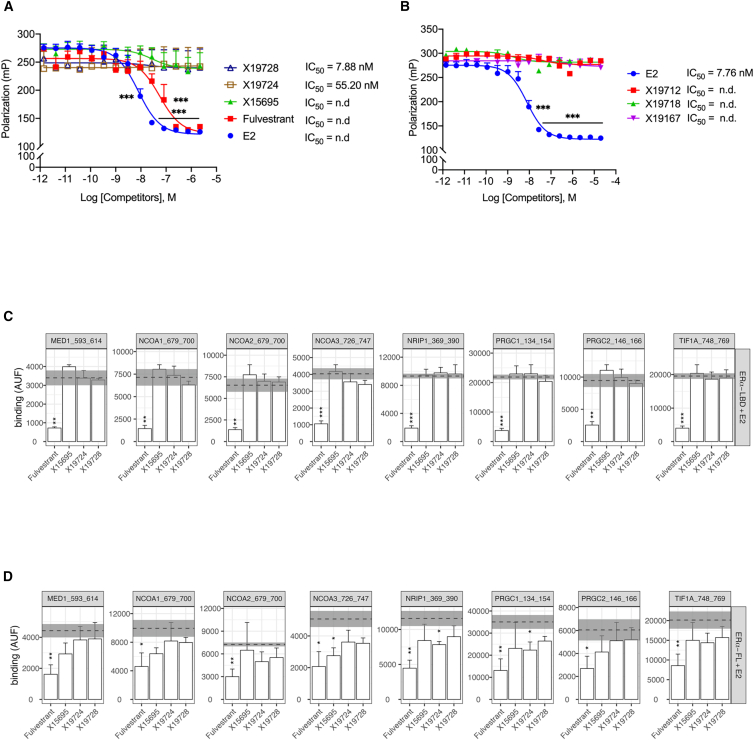
Imidazopyridines inhibit breast cancer cell proliferation independent of direct ERα binding (see also Figure S1). Competitive binding data generated using the PolarScreen ERα Competitor Assay. (A and B) Polarization values are plotted against the concentration of the test compound. Data were modeled using GraphPad Prism software from GraphPad Software, Inc. The results represent the mean values ± SEM. Statistical significance was assessed by multiple *t* test (*n* = 2–8; ∗∗∗*p* < 0.001). (C and D) Compound effect on E_2_-induced ER⍺-coregulator interaction, using the NAPing platform (PML, Oss, The Netherlands), zooming in on a subset of (well-established) ERα-coregulators: mediator of RNA polymerase II transcription subunit 1 (MED1); nuclear receptor coactivator −1, −2, −3 (NCOA-1, -2, -3); nuclear receptor-interacting protein 1 (NRIP1); peroxisome proliferator-activated receptor gamma coactivator 1a (PRGC1); peroxisome proliferator-activated receptor gamma coactivator 1b (PRGC 2), and transcription intermediary factor 1-alpha (TIF1A). Recombinant ER⍺ LBD (C) or ER⍺ FL in MCF-7 extracts (D) was treated with E_2_ EC_50_ (−8.5 logM) and subsequently incubated with 1000-fold excess (−5.5 logM) of each compound. Binding is represented as mean arbitrary units of fluorescence of the detection antibody (AUF) ± SEM (error bar in test compounds and gray area for control) of three technical replicates per indicated condition. Significance of test compound-induced modulation of E_2_-bound ER control binding was assessed using Student’s *t* test (∗*p* < 0.05; ∗∗< 0.01; ∗∗∗< 0.001).

To explain the observed differences in the action of the imidazopyridine compounds and fulvestrant toward ER^+^ breast cancer cells, we determined the relative binding affinities of imidazopyridines (X15695, X19724, and X19728) and fulvestrant to the ERα using a polar screen competition assay. This assay determines the effectiveness of ligands to compete with a selective fluorescent Fluormone tracer for binding to ERα. ERα and the Fluormone tracer form a complex, resulting in a high fluorescence polarization value. Compounds that bind ERα displace the Fluormone tracer from the complex and cause a decrease in fluorescence polarization. As expected, both estradiol and fulvestrant efficiently displaced the Fluormone tracer from the ERα/Fluormone tracer complex. However, the three imidazopyridine derivatives did not (Figure 3A), indicating that they do not associate with the ERα. Other imidazopyridines (X19712, X19718 and X19167) (Figure 1A) from our collection of 2-phenylimidazo[1,2-*a*] pyridines that did not inhibit ER^+^ breast cancer cell proliferation^17^ also failed to displace the Fluormone (Figure 3B).

We next determined whether the imidazopyridines function as orthosteric antagonists by disrupting coactivator recruitment to the ERα. We used an assay that measures nuclear receptor (NR) binding to a collection of coregulator-derived interaction motifs *in vitro*, and thereby mimics the recruitment of proteins that play a role in the canonical regulation of transcriptional activity. The estradiol (E_2_)-activated ERα LBD or a full-length ERα was incubated with 101 peptides of well-known NR coregulators immobilized on a solid support, and the ability of the imidazopyridine compounds to disrupt the binding was determined at a single dose. Fulvestrant, used as a control in this assay, clearly disrupted binding of the coactivator peptides to either the liganded LBD or full-length ER in a manner consistent with ER antagonism as previously reported^27^ (Figures 3C, 3D, S1A, and S1B). In contrast, none of the imidazopyridine compounds analyzed showed this activity (Figures 3C, 3D, S1A, and S1B). These data support our hypothesis that the imidazopyridine derivatives do not directly target ERα.

**Figure 4:**
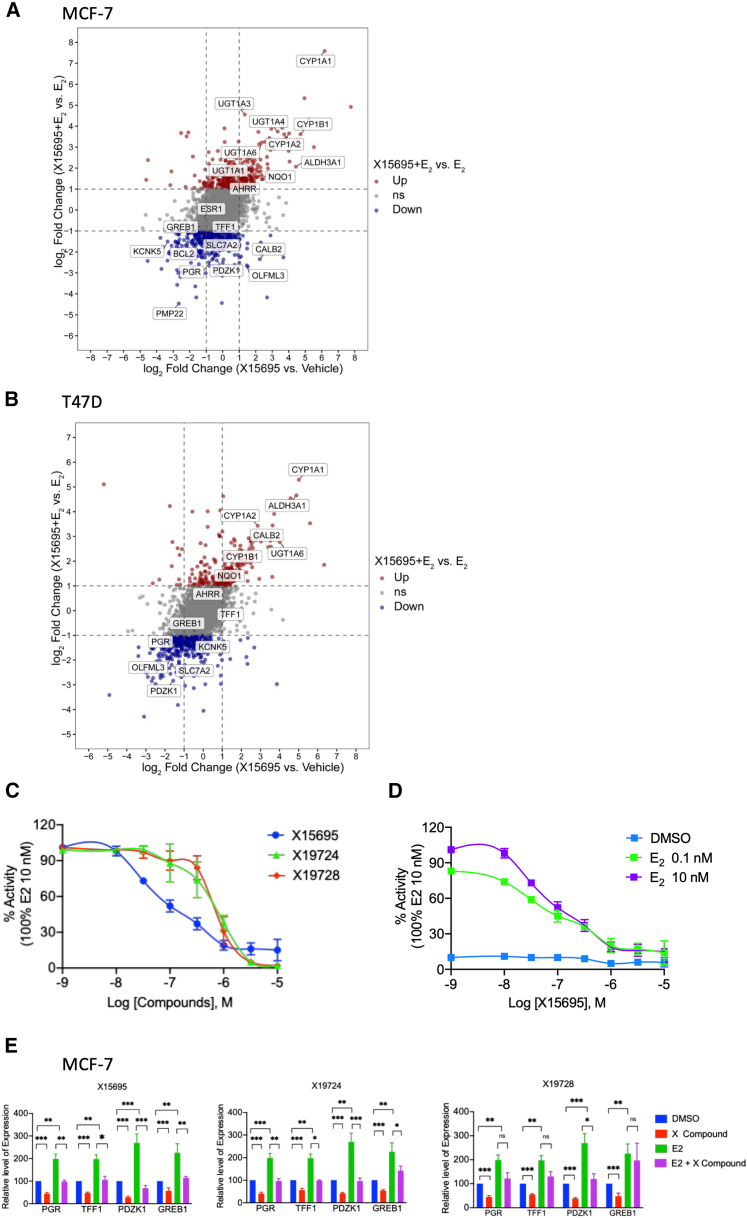
Attenuation of estrogen receptor action by the imidazopyridines (see also Figure S2). (A and B) Four-way plots show differentially expressed genes (DEGs) in MCF-7 cells (A) and T47D cells (B) treated with X15695 under estrogen-deprived condition (X15695 vs. vehicle) (X axis) or E_2_-supplemented condition (X15695 + E_2_ vs. E_2_) (Y axis). Genes with |Log_2_(Fold Change)| ≥ 1 and adj. *p*-value ≤0.05 were considered significant. Upregulated and downregulated DEGs are highlighted in red and blue, respectively; non-significant genes are shown in gray. Select representative ER target genes (blue) and AHR target genes (red) are explicitly labeled. In the 4-way plot, each gene has a coordinate (X,Y), the X value shows the log_2_FC of X15695 vs. vehicle, while Y value refers to the log_2_FC of X15695_E_2_ vs. E_2._ (C and D) Results of reporter gene assay on the action of the indicated imidazopyridines on the transactivation function of the ERα. Cells were hormone starved for 24 h and treated with the indicated compounds (10 nM–10 μM) in the presence of 10 nM (C) or 0.1 or 10 nM (D) E_2_ for 24 h. The results are the mean value ± SEM of independent biological replicates (n = 2–4). (E) Quantitative RT-PCR to detect the effect of the imidazopyridine derivatives on the expression of the indicated ER target genes in MCF-7 cells in the presence and absence of E_2_. Cells were serum starved for 72 h and treated with the indicated compounds (1 μM) in the presence and absence of 10 nM E_2_ for 24 h. The data represent the mean ± SEM. Statistical significance was assessed by multiple *t* test (*n* = 3; ∗*p* < 0.05; ∗∗< 0.01; ∗∗∗< 0.001; ns is not significant).

Despite this lack of evidence for direct ERα interaction, our previous RNA-seq and RT-PCR studies showed X15695 attenuated ERα target gene expression in MCF-7 and T47D cells.^17^ We therefore reassessed these datasets and showed in 4-way plots that some of the strongest differentially expressed genes (DEGs) with X15695 are indeed ER target genes (Figures 4A and 4B). Using a cell-based luciferase ER/ERE reporter assay, the transcription activity of ERα was also reduced by X15695, X19724, and X19728 (Figures 4C and 4D), albeit X19724 and X19728 actions were somewhat weaker (Figure 4C). RT-PCR studies were also carried out with the three compounds on a select number of the ERα target genes (*PGR*, *TFF1*, *PDZK1,* and *GREB1*) identified in the 4-way plots. This assay confirmed that all three compounds downregulated the expression of ERα target genes, both in the absence and in the presence of E_2_ (Figure 4E), although the action of X19728 was not significant in the presence of E_2_.

### The aryl hydrocarbon receptor is the direct molecular target of antiproliferative imidazopyridines

We hypothesized that since the imidazopyridines do not bind ERα, they most likely attenuate ERα gene expression indirectly, possibly through association with a component that in turn downregulates ERα activity. Our previous RNA-seq experiments carried out in MCF-7 and T47D cells after treatment with E_2_ and X15695 identified xenobiotic metabolism as one of the topmost signaling pathways in the gene set enrichment analysis (GSEA).^17^ Heatmaps of the Log_2_ fold-change in gene expression in X15695 vs. vehicle or E_2_ + X15695 vs. E_2_ treatment in MCF-7 and T47D cells identified several genes involved in xenobiotic metabolism (Figures S2A and S2B). Importantly, several of the upregulated genes are AHR target genes (Figures 4A and 4B). The AHR is known to interact with and to alter the transcriptional activity of, among others, the estrogen and androgen receptors (ARs).^28^ We therefore postulated that our imidazopyridine derivatives possibly function by interacting with the AHR.

**Figure 5:**
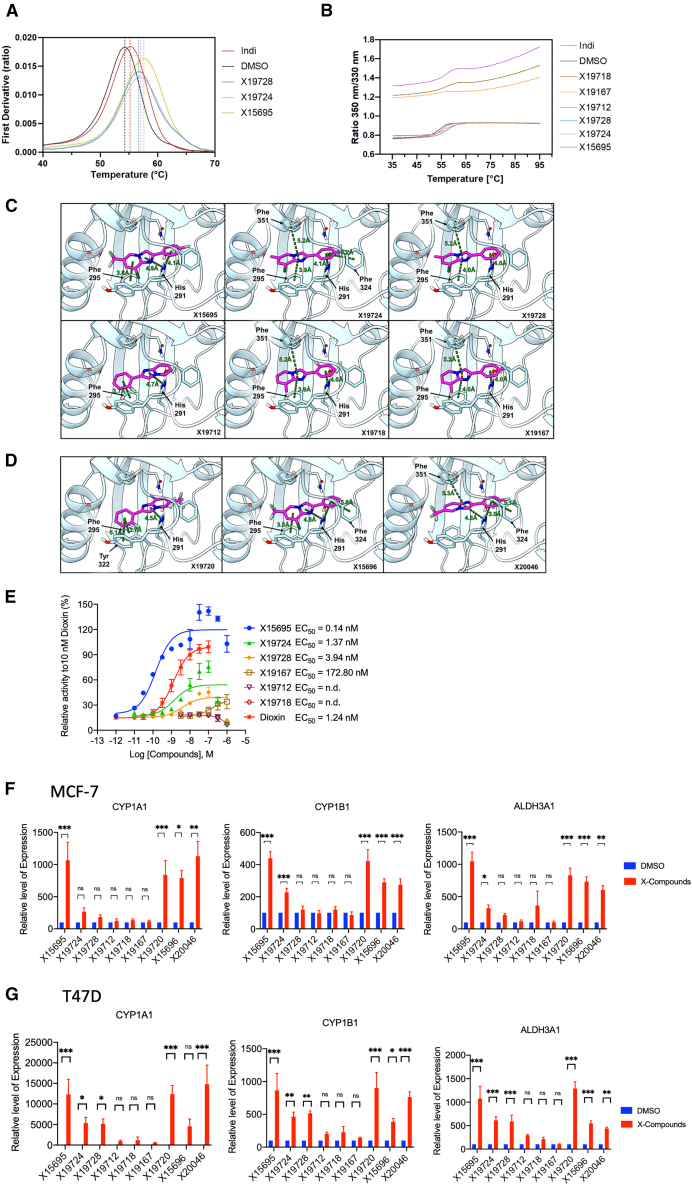
The aryl hydrocarbon receptor is the direct molecular target of antiproliferative imidazopyridines (A and B) Nano-DSF analysis of the interaction between Hsp90-XAP2-AHR complexes and the indicated imidazopyridines. DMSO and Indirubin (indi) were used as negative and positive controls. The results are representative of independent biological replicates (*n* = 3). The results in (A) show the first derivative plot for X15695, X19724, and X19728. In (B) are the fluorescence ratio plots (F350/F330) as a function of temperature that show the raw protein unfolding transition. (C and D) Results of molecular docking calculations. The binding modes of six imidazopyridine derivatives (X15695, X19724, X19728, X19712, X19718, and X19167) and (D) three additional imidazopyridines (X19720, X15696, and X20046) within the PAS-B binding domain of AHR shown in a three-dimensional representation: ligands and the main interacting residues are shown as sticks, protein as white cartoons; π- π stacking interactions, as identified by the ligand interaction diagram (Maestro, Schrödinger), are represented as green dashed lines; the interacting residues and the corresponding centroid-to-centroid distances are labeled. (E) Results of reporter gene assay on the action of the indicated imidazopyridines on the transactivation function of the AHR. HAHLH cells were serum starved for 24 h and treated with the indicated concentrations of the compounds for 8 h. Results were expressed as % of the maximal luciferase activity obtained with dioxin 10 nM. The results are the mean value ± SEM of independent biological replicates (n = 2–4). (F and G) Quantitative RT-PCR to detect the effect of the indicated imidazopyridine derivatives on the expression of three AHR target genes in MCF-7 and T47D cells. Cells were serum starved for 72 h and treated with the indicated compounds (1 μM) for 16 h. The data represent the mean ± SEM. Statistical significance was assessed by multiple *t* test (n = 3–12; ∗*p* < 0.05; ∗∗< 0.01; ∗∗∗< 0.001; ns is not significant).

Differential scanning fluorimetry (nano-DSF) monitors the thermal stabilization of proteins upon ligand binding, and we used this to confirm that the imidazopyridines bound purified AHR-Hsp90-XAP2. Indeed, they stabilized the AHR to a greater degree than the classical AHR ligand, indirubin (Indi), which was used as a positive control in these assays (Figure 5A). The ranking order was X15695 > X19724 > X19728 (Figure 5A). The raw data show a low initial fluorescence ratio and a clear unfolding event that reaches a plateau at the highest temperatures (Figure 5B). Other 2-phenylimidazo[1,2-*a*] pyridine derivatives X19167, X19712, and X19718 (Figure 1A), which poorly inhibited breast cancer cell proliferation,^17^ displayed very different profiles with a high initial ratio, suggesting partial or complete protein unfolding in the presence of these ligands (Figure 5B). Strikingly, an unfolding inflection could still be observed in the presence of X19712 and X19718, but not with X19167. These observations strongly suggest that these latter three molecules interact with AHR, either specifically or nonspecifically, and that such interactions lead to varying degrees of receptor destabilization. We therefore identified X15695, X19724, and X19728 as *bona fide* AHR ligands, in contrast to X19167, X19712, and X19718, which appear to promote AHR destabilization.

To reveal how the imidazopyridines bind to the AHR, we used *in silico* docking calculations that allow detailed insight into the binding modes and potential binding affinities of the compounds. For this, we used the relevant human experimental structures of the AHR PAS-B domain (PDB IDs 7ZUB) in complex with indirubin.^29^ We utilized the Glide software, a part of the Schrödinger suite, for all docking calculations. As represented in Figure 5C, most compounds formed a π-π stacking interaction network (represented by green dashed lines). This network was primarily anchored by a stacking interaction between the imidazopyridine ring of the ligands and the aromatic side chain of Phe295, with a T-stacking further stabilizing the secondary phenylic ring. Additional π−π stacking interactions were observed with Phe351 and Phe324. The docking scores showed that X15695 has the highest predicted affinity (the most negative score) while X19724 and X19728 exhibited slightly lower affinities (Table 1). The remaining three ligands that did not inhibit ER^+^ breast cancer proliferation had the lowest predicted binding affinities (less negative docking scores). These results agree with the nano-DSF study that X15695 is the compound with the strongest affinity for the AHR.

**Table 1:** Glide score and predicted pKa of the different imidazopyridine compounds

Compounds	Inhibition of Proliferation	Glide XP Score (kcal mol^−1^)	Predicted pKa
X15695	Yes	−10.13	3.09
X19724	Yes	−9.357	4.50
X19728	Yes	−8.791	4.69
X15696	Yes	−10.055	3.28
X19720	Yes	−10.377	3.28
X20046	Yes	−9.76	4.83
X19712	No	−8.354	6.27
X19718	No	−8.608	6.55
X19167	No	−8.65	6.36
Imidazo(1,2-*a*)pyridine	–	–	6.69

To complete the AHR binding analysis for all six imidazopyridines previously identified as potent cell proliferation inhibitors^17^ (Figure 1A), docking calculations were also performed on the remaining three compounds in that group (X19720, X15696, and X20046). These three compounds exhibited docking scores comparable to that of X15695 (Table 1), indicating high binding affinities for the receptor. Also, their binding modes, predicted by docking calculations (Figure 5D), were like those previously observed for the other inhibitors studied in this work (Figure 5C).

Intriguingly, during the preparation phase of the compounds, we noted that the aqueous phase pKa values (predicted by the *Epik* tool) present important differences among the compounds (Table 1). All six experimentally active compounds that inhibited breast cancer cell proliferation (X15695, X19724, X19728, X15696, X19720, X20046) were predicted to be almost exclusively neutral at physiological pH, with pKa values for the imidazopyridine nitrogen ranging from 3.5 to 4.8. In contrast, all experimentally inactive compounds (X19712, X19718, X19167) had a pKa close to 6.5, implying they exist as an equilibrium mixture of neutral (∼89%) and protonated (∼11%) forms. As a control, we also predicted the pKa for the non-functionalized 2-phenylimidazo[1,2-*a*]pyridine compound, obtaining a value of 6.69, which aligns well with the experimental value of 6.79 reported in the IUPAC Digitized pKa Dataset,^30^ thus confirming the reliability of our approach. Two effects may contribute to the observed behavior of differently substituted imidazopyridine rings. On the one hand, the electron-withdrawing substituents that lead to a low pKa also render the aromatic system electron-deficient, enhancing the crucial π-π stacking interactions with key residues such as Phe295 and His291, as supported by our docking calculations. On the other hand, a high pKa is detrimental to binding affinity. This might seem counterintuitive, as the resulting protonated cation would be expected to form even stronger cation-π interactions. However, the predominantly hydrophobic nature of the pocket imposes a large energetic penalty for the desolvation of a charged species, making the binding of the protonated form highly unfavorable from a thermodynamic point of view. Therefore, we propose that potent AHR ligands must first exist in a neutral state to efficiently partition into the binding site. This condition is met only by the compounds with low pKa values, which combine favorable neutrality with an electronic profile optimized for π-π stacking.

### Imidazopyridines act as potent agonists of the AHR signaling pathway

To determine whether ligand interaction with the AHR correlates with functional activity, we first analyzed the AHR transactivation function in a cell-based luciferase reporter gene assay after the treatment of the cells with the six 2-phenylimidazo[1,2-*a*] pyridine compounds (Figure 1A). Of note, X15695 upregulated the transcriptional activity of the AHR with a potency that surpassed that of the classical AHR ligand dioxin (Figure 5E). X19724 and X19728, however, showed only modest enhancement, with marginal effects both in terms of potency and efficacy (Figure 5E). The other imidazopyridine compounds that showed reduced docking scores were functionally inactive in the reporter gene assay at the concentrations tested (Figure 5E).

We also performed RT-PCR gene expression analysis for the three AHR target genes *CYP1A1*, *CYP1B1,* and *ALDH3A1* using all the imidazopyridine compounds (Figure 1A). This was done in both MCF-7 (Figure 5F) and T47D cells (Figure 5G). A gene and cell line-selective effect was evident in the action of the compounds X19724 and X19728 (Figures 5F and 5G), consistent with reports on the behavior of AHR ligands.^31^ In contrast, X15695, X15696, X19720, and X20046, the sub-group with the C-6 CF3 substitutions, were more consistent in their effects and enhanced the transcriptional activity of the AHR target genes in both MCF-7 and T47D cells (Figures 5F and 5G). Compounds X19712, X19718, and X19167 that poorly inhibited breast cancer cell proliferation failed to activate the expression of the AHR target genes in the two cell lines (Figures 5F and 5G). The active imidazopyridines X15696, X15695, and X19720 have marginal thermodynamic solubilities of 5 μM, 3 μM, and 1.4 μM, respectively, as determined using the shake flask method (Table S1). They were nonetheless remarkably stable over 5 days at 21 °C with no obvious decomposition detected (Figure S3). These findings need to be taken into consideration in future development and formulations of these compounds for therapeutic applications. In a parallel artificial membrane permeability (PAMPA) assay^32^ (also see STAR Methods), X15695, the most active member of the imidazopyridines studied, showed an apparent permeability coefficient (P_app_) of 8.68×10^−6^ cm s^−1^, which is comparable to the relatively well-permeable compound, caffeine (P_app_ = 10.7×10^−6^ cm s^−1^) (Table S2). This is a key finding in the potential oral bioavailability of this compound.

### The antiproliferative effect of imidazopyridines is strictly dependent on the AHR

**Figure 6:**
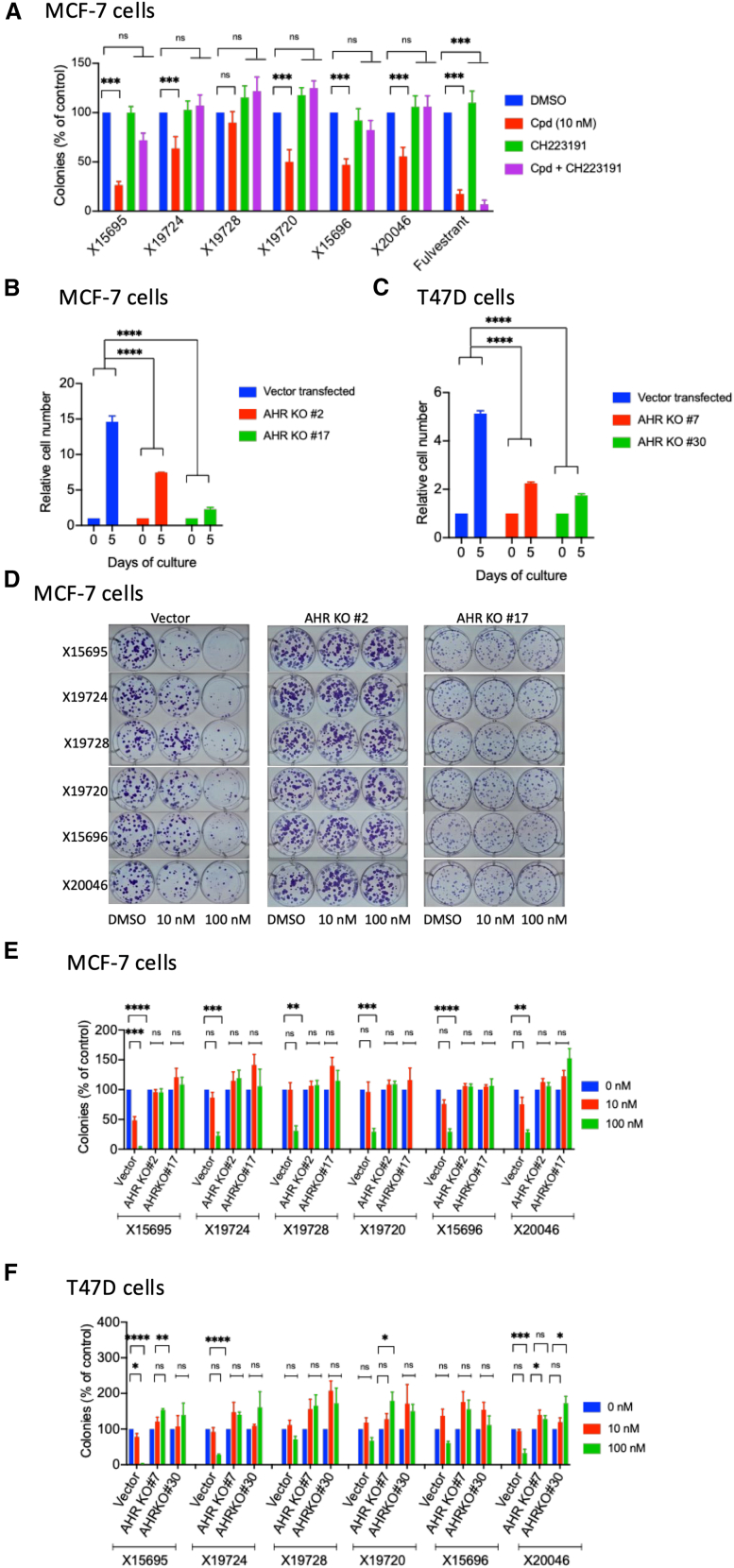
The antiproliferative effect of imidazopyridines is strictly dependent on the AHR (see also Figure S4). (A) Effect of the AHR antagonist CH223191 on the inhibition of clonal expansion by the X compounds. Quantification of the action of the AHR antagonist CH223191 on the inhibition of the clonal expansion of MCF-7 cells by the indicated X compounds. Cells were treated with 10 nM of the X compounds in the absence and presence of 1 μM CH223191 for 14 days. The values are the means ± SEM. Statistical significance was assessed by multiple *t* test. (*n* = 4. ∗∗∗*p* < 0.001; ns is not significant). (B and C) Proliferation of empty vector-transfected MCF-7 and T47D cells, as well as MCF-7 AHR KO clones #2 and #17, and T47D AHR KO clones #7 and #30. Cells were cultured in complete media and counted on days 0 and 5. The starting number of cells was 1 ×10^4^ per well. Data are the averages of three independent experiments ± SEM, normalized to day 0. Statistical significance was assessed by two-way ANOVA (∗∗∗∗*p* ≤ 0.0001). (D) Representative images of the clonal expansion of empty vector-transfected MCF-7 cells or AHR KO MCF-7 cell clones #2 and #17 after treatment with the indicated concentrations of X compounds. (E) Quantification of the concentration-dependent action of the indicated compounds on the clonal expansion of empty vector-transfected MCF-7 cells and AHR KO MCF-7 clones #2 and #17. The results are shown as bar charts, and they represent the means ± SEM. Statistical significance was assessed by one-way ANOVA (n = 3–4. ∗∗∗*p* < 0.001; ∗∗∗∗ <0.0001; ns is not significant). (F) Quantification of the concentration-dependent action of the indicated compounds on the clonal expansion of empty vector-transfected T47D cells and AHR KO T47D clones #7 and #30. The results are shown as bar charts, and they represent the means ± SEM. Statistical significance was assessed by one-way ANOVA (n = 3–4. ∗*p* < 0.05; ∗∗*p* < 0.01; ∗∗∗*p* < 0.001; and ∗∗∗∗*p* < 0.0001; ns is not significant).

Having established the features of the imidazopyridines that contribute to AHR binding, we next sought to correlate this binding to the inhibition of ER^+^ breast cancer cell proliferation. For this, we performed clonogenic assays in MCF-7 cells and blocked AHR activity with CH223191.^33^ Except for X19728, which had no effect on colony formation (Figure 6A), the inhibition of colony expansion by the compounds X15695, X15696, X19720, and X20046 (and, to a lesser extent, X19724) was clearly abrogated by the AHR antagonist CH223191 (Figure 6A, compare red with purple bars). Fulvestrant inhibited colony growth in the presence of CH223191, demonstrating its independence from AHR and a different mode of action for the inhibition of ERα-dependent breast cancer cell proliferation.

As the competitive inhibitor CH223191 leaves the AHR intact and only blocks its activity, we also generated a complete AHR knockout (KO) in MCF-7 and T47D cells using CRISPR technology that permanently removes AHR from cells (Figure S4A). Two MCF-7 (#2 and #17) and T47D (#7 and #30) AHR KO clones (Figures S4B and S4C) showed decreased proliferation compared to the empty vector-transfected cells, demonstrating that the AHR contributes to the proliferation of these cells (Figures 6B and 6C). When treated with the imidazopyridines, the MCF-7 AHR KO clones became resistant to the inhibition of colony growth that is normally observed in the control vector-transfected cells (Figures 6D and 6E). These findings agree with the results obtained following AHR inhibition with CH223191 (Figure 6A), showing an even stronger effect upon the CRISPR-mediated ablation of AHR. This confirms that the imidazopyridines act via the AHR to reduce breast cancer cell proliferation. The inhibitory action of X19728 in MCF-7 cells was again less pronounced compared to X15695 (Figures 6D and 6E). Similar results were obtained in T47D KO clones (Figure 6F), but, overall, the T47D cells were less sensitive than MCF-7 cells to inhibition by the X compounds. In both cell types, X15695 was the most effective in reducing cell proliferation.

### Antiproliferation effect of imidazopyridines is independent of ERα turnover

**Figure 7:**
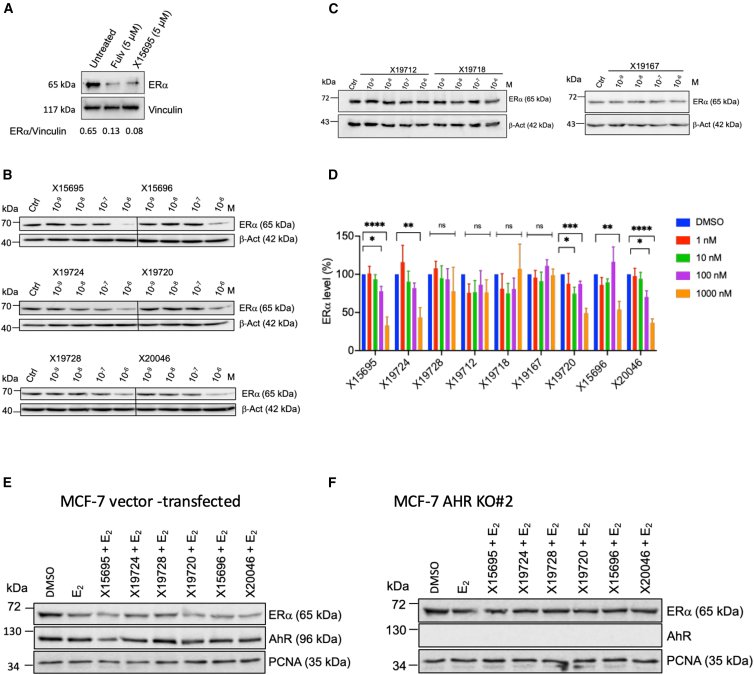
Antiproliferation effect of imidazopyridines is independent of ERα turnover (A) Immunoblot analysis of ERα in PDxO #28 treated with vehicle (untreated) or fulvestrant or X15695 for 48 h as indicated. Vinculin was used as a loading control. 7 μg protein was used for loading in each lane. Below is the ratio between ERα and vinculin calculated for each condition. (B and C) Representative western blots show the level of ERα expression after the treatment of MCF-7 cells with the indicated concentrations of imidazopyridine compounds for 48 h. An anti-ERα antibody was used to detect the ER signal, and an anti-β-actin antibody was used for the loading control. 30 μg protein was loaded in each lane. Six replicates were analyzed in (B), while 50 μg protein was loaded in each lane in (C), and three replicates were analyzed. (D) Protein signals in the western blots in (B) and (C) were quantified. The bar charts show the level of ERα expression relative to the β-actin expression, and they represent the means ± SEM. Statistical significance was assessed by one-way ANOVA (*n* = 3–6; ∗*p* < 0.05; ∗∗< 0.01; ∗∗∗< 0.001; and ∗∗∗∗< 0.0001; ns is not significant). (E and F) Requirement of AHR for the destabilization of ERα by the indicated imidazopyridines. MCF-7 cells (empty vector transfected) (E) and MCF-7 AHR KO clone #2 (F) were cultured in hormone-deprived medium for 72 h and treated for 8 h with E_2_ (10 nM) or E_2_ + the indicated X compounds (1 μM). Western blots were carried out with 50 μg protein lysates of these cells using anti-ERα and AHR antibodies, along with anti-PCNA antibody to demonstrate equal protein loading. The Western blots are a representative example of 3 experiments that produced similar results.

Among the many effects of X15695 on different signaling molecules that we have previously described,^17^ is its ability to decrease ERα stability. Immunoblot analysis of ERα levels in organoids PDxO#28 (Figures 2D and 2E) indeed demonstrated that X15695 degraded ERα similar to or stronger than that seen with fulvestrant (Figure 7A). However, it is unclear whether ERα destabilization is causative for the growth inhibition properties of the imidazopyridines. We therefore treated MCF-7 cells for 48 h with increasing concentrations of all six imidazopyridines that we previously identified as inhibitors in our earlier study (namely X15696, X19720, X15695, X20046, X19728, X19724), in addition to the compounds X19712, X19718 and X19167 that we identified as poor inhibitors of breast cancer cell proliferation (Figure 1A).^17^ Analyses of their ability to degrade ERα using an immunoblot assay identified five compounds (X15695, X19724, X19720, X15696 and X20046) as being the most proficient in destabilizing ERα (Figures 7B and 7C). X19728, such as the compounds X19712, X19718, and X19167 that poorly inhibited breast cancer cell proliferation,^17^ showed no significant ERα destabilization at the concentrations used in the study (Figures 7B and 7C). While these results appear to link ERα destabilization with the anti-proliferative action of the compounds, the concentrations required for the ERα destabilization are higher compared to those required for the anti-proliferation activity. Destabilization occurred at 1 μM or at best at 0.1 μM, while proliferation inhibition occurred in the nanomolar range in the clonogenic assay^17^ (Figures 1B and 1D). Furthermore, while X19728 inhibited the proliferation of parental MCF-7 cells at 100 nM (Figures 6D and 6E), no destabilization of ERα was observed even at 1 μM concentration (Figures 7B and 7D). This demonstrates that the destabilization of ERα does not directly account for the anti-proliferative action of this compound.

We then questioned whether the AHR dependency of the imidazopyridines was required for the destabilization of ERα by comparing the action of the six ligands in the empty vector-transfected MCF-7 and the MCF-7 AHR KO cells after treatment with E_2_ and E_2_ + X-compounds. While E_2_ alone, as expected, degraded ERα^34^ (Figure 7E), E_2_ together with the imidazopyridines showed a much clearer destabilization in the vector-transfected but not in the AHR KO cells (Figures 7E and 7F), consistent with the affinity of the compounds for the AHR. Combined, these data demonstrate that the imidazopyridine-induced destabilization of ERα is linked to the affinity of the compounds for the AHR but is insufficient to account for the anti-proliferative action of the compounds.

## Discussion

The ERα is a critical target of therapeutic significance in the control of ER^+^ breast cancer. However, the clinically available ER antagonists used for the treatment of breast cancer have limitations, due, in part, to mutations in the ERα gene (*ESR1*) that make the tumors intractable to treatment. SERDs have therefore been developed to overcome these problems but fulvestrant, the first clinically available SERD, showed only modest inhibition of mutant ERα action compared to wildtype ERα.^3^ Therefore, other SERDs with improved mutant ERα inhibition have since been developed or are in development.^12^

We have previously described the compound X15695, a derivative of 2-phenylimidazo[1,2-*a*] pyridine that inhibits ER^+^ breast cancer proliferation, and which we classed as a SERD because it was found to degrade ERα. In this study, we showed that in the presence of estradiol, a condition that mimics the pre-menopausal situation, X15695 and additional derivatives outperformed fulvestrant in inhibiting the proliferation of breast cancer cells that possess wild-type and mutant ERα. Additionally, the ability of X15695 to outperform fulvestrant was confirmed in several patient-derived breast cancer models, further supporting the clinical relevance of this compound. As one of the characteristic features of SERDs is direct binding to ER,^35^ we determined whether the imidazopyridine compounds in our study bind ERα.

As opposed to fulvestrant, these imidazopyridines did not interact with ERα nor did they inhibit the NR-box (LxxLL)-mediated recruitment of coactivators that are otherwise required for ERα agonism or targeted by most conventional SERDs or SERMs.^36^ Instead, the compounds interacted with the AHR. Pharmacological inhibition of the activity of the AHR or genetic ablation of the AHR attenuated the inhibitory action of the compounds on breast cancer cell proliferation, demonstrating that their anti-cancer effects are dependent on functional AHR in cells.

AHR is a member of the basic helix-loop-helix-PER-ARNT-SIM (bHLH-PAS) family of transcription factors. It carries a conserved bHLH DNA binding domain at its N-terminus, followed by tandem PAS domains (PAS-A and PAS-B), and a transactivation domain.^37^ The PAS-B domain functions as the primary binding site for small-molecule ligands ranging from polycyclic aromatic hydrocarbons to polychlorinated biphenyls, but the precise basis for the interaction of these diverse ligands has until recently remained elusive.^31^ The crystal structures of the AHR in complex with the aryl hydrocarbon receptor nuclear translocator (ARNT) bound to DNA and each of six classical AHR ligands have now been solved. This revealed that for transcriptional activity, the PAS-B domain utilizes eight conserved residues arranged in such a way that it binds to ligands through hydrophobic and π-π interactions.^31^

Apart from the regulation of transcription, liganded AHR has non-genomic actions such as binding to and degrading transcription factors such as the ERα and the AR.^38^^,^^39^^,^^40^^,^^41^^,^^42^ However, the features on the ligands that determine these effects have remained elusive. While some AHR ligands induce ERα degradation, other ligands negatively regulate activity independent of degradation, whereas yet other ligands activate ERα signaling.^43^^,^^44^ Ligands including 2,3,7,8-tetrachlorodibenzo-*p*-dioxin, 6-formylindolo(2,3 b) carbazole, carbidopa, and the prenylflavone icaritin^38^^,^^39^^,^^40^^,^^41^^,^^42^^,^^45^ have been described to endow the AHR with the ability to degrade ERα.

The exact mechanism of AHR-mediated destabilization of NRs or other proteins is not clear. One possibility is that the AHR acts as a substrate-recognition subunit to recruit ERα/AR for proteolysis by assembling a ubiquitin ligase complex, CUL4B(AHR).^38^^,^^46^^,^^47^ The involvement of another E3 ligase has also been reported. For example, indole-3-carbinol-dependent activation of AHR has been shown to initiate the E3 ubiquitin ligase ring box 1 (Rbx-1) and proteasomal degradation of ERα.^48^ Evidence that E3 ligase complexes recruited by the AHR bring about protein degradation comes from recent studies that show that the incorporation of AHR ligands into proteolysis-targeting chimeras (PROTACs) generates molecules for the targeted degradation of cellular proteins.^49^ Other proposed putative actions of AHR-mediated inhibition of ERα signaling include the attenuation of ER target gene expression by the direct binding of the activated AHR and ARNT heterodimer complex to inhibitory xenobiotic response elements (iXREs) in ER target genes. Alternatively, “squelching” of AHR coactivators, including ARNT, and synthesis of an unknown inhibitory protein have been proposed.^50^

In our previous study, we showed that X15695 reduced ERα levels.^17^ Here, we show that this activity is shared by distinct 2-phenylimidazo[1,2-*a*] pyridine derivatives that strongly bind the AHR. However, we noted that the concentrations of the imidazopyridines required for ERα degradation are a lot higher than those required for their anti-cancer effect, indicating that ERα degradation is not causally linked to the anti-proliferative effects of the compounds. Instead, our previous findings that X15695 increased oxidative stress, p53 target gene expression, and cell-cycle arrest and apoptosis^17^ may contribute to the antiproliferative action of the imidazopyridines. Intriguingly, some of these effects were also observed in prostate cancer cells treated with X15695.^17^ Although the inhibition of prostate cancer cell proliferation is not the subject of this investigation, it is important to mention that we previously observed that X15695-mediated antiproliferative action was partly abrogated by p53 siRNA knockdown, not only in breast cancer but also in prostate cancer cells,^17^ confirming a contribution of p53 to the action of the imidazopyridines. An interplay of p53 and AHR signaling pathways has previously been reported by the group of Kolluri where they showed that the loss of AHR and p53 together increased tumorigenesis in mice,^51^ albeit the mechanistic details remain to be clarified. Future studies will be needed to decipher the intricacies of the crosstalk of these two signaling pathways.

In our current study, we showed that the imidazopyridines do not bind ERα, and as selective ER binding is one of the characteristic features of SERDs, causing the receptor to be degraded and inhibiting cancer cell growth,^52^ the imidazopyridines described here cannot be classified as SERDs. We observed that the inhibitory action of the imidazopyridines is selective for ER^+^ over ER^−^ breast cancer cells,^17^ which is likely due to a defect in the action of the AHR in the ER^−^ cells rather than the absence of ER expression. The AHR is constitutively expressed and does not respond to exogenous ligands^53^ in the MDA-MB231 ER^−^ breast cancer cell line used in our studies. A similar explanation may be given to the lack of antiproliferative action of the imidazopyridines in our previous studies in AR^−^ PC3 and DU145 over AR^+^ LNCaP prostate cancer cells.^17^ Like the ER^−^ MDA-MB231 breast cancer cells, the AR^−^ prostate cancer cells express high levels of AHR and do not respond to treatment with AHR ligands.^54^ As the expression of the AHR is widespread across a variety of cancer types,^55^ future therapeutic development of the imidazopyridines should not be limited to breast cancer but extended to other tumor types.

One of the key findings in the present study is the link between the anti-proliferative effect of 2-phenylimidazo[1,2-*a*] pyridine derivatives and their affinities to the AHR. We have shown that not all the imidazopyridines that bind the AHR inhibit breast cancer cell proliferation. We used computational docking experiments to determine which ligands inhibit ER^+^ breast cancer cell proliferation and bind the AHR and which do not. We noted that 2-phenylimidazo[1,2-*a*] pyridine derivatives that strongly interact with the AHR most potently inhibit ER^+^ breast cancer cell proliferation. These compounds were predicted to have low pKa values, suggesting the presence of electron-withdrawing substituents that deplete electron density from the aromatic ring. Decreasing the electron density in the π-cloud causes a reduction of the electrostatic repulsion between π-systems, thereby enhancing the π-π stacking interactions as has been described in several theoretical models.^56^^,^^57^^,^^58^ Thus, it is conceivable that ligands bearing electron-withdrawing substituents exhibit enhanced AHR affinity due to an extra-stabilization of the π-stacking interactions with the aromatic residues in the binding cavity, particularly with F295. These properties are clearly identifiable in the four compounds of this study (X15695, X15696, X19720, and X20046), which may need further development for therapeutic application.

### Limitations of the study

Our studies clearly show that distinct 2-phenylimidazo[1,2-*a*] pyridine derivatives interact with the AHR and that the AHR mediates the inhibition of ER^+^ breast cancer cell proliferation by these compounds. However, there are certain limitations to our study. The exact mechanism for the inhibition of cell proliferation by the AHR is unknown. It does not seem to be purely dependent on the degradation of ER^+^ that was observed in this work, as other signaling pathways, such as p53, appear to contribute to the anti-proliferative activity of AHR in the ER^+^ breast cancer cells. Furthermore, because of the pleiotropic biology of AHR, systemic activation of this receptor may have complex immunological and/or toxicological effects, and these need to be ruled out for the future therapeutic exploitation of our findings.

## Resource availability

### Lead contact

Further information and requests for resources and reagents should be directed to and will be fulfilled by the Lead Contact, Andrew C. B. Cato (andrew.cato@kit.edu).

### Materials availability

This study uses 2-phenylimidazo[1,2-*a*] pyridine derivatives targeting the AHR. Protocols and related research data for the synthesis of the compounds X15695, X19724, X19728, X19712, X19718, X19167, X19720, X15696, and X20046 can be accessed through the data repository Chemotion (Gräßle, S. *Chemotion Repository*. (2023), Available online: https://doi.org/10.14272/collection/SGV_2022-09-29). Samples have been deposited in the Molecule Archive (https://compound-platform.eu/) and can be requested from there. CRISPR/Cas9 KOs of AHR were also generated in MCF-7 and T47D cells. These materials will be available for academic researchers upon completion of the Material Transfer Agreement.

### Data and code availability

• The RNA-seq data are deposited at the GEO repository under accession number GSE218556.
• This paper does not report original code.
• Any additional information required to reanalyze the reported data is available from the lead contact upon request.

## Acknowledgments

We thank Carsten Weiss for fruitful discussions and suggestions. We acknowledge with thanks the help we received from Celine Moser and Claudia Muhle-Goll with the use of the Tecan Reader for the fluorescence polarization studies. M.P. received funding for his contribution to this work from the 10.13039/501100004543China Scholarship Council (CSC), grant no. 201807090113; B.B. received funding from the Ministry of Health (RF-2021-12371961) and AIRC (IG20061). This project was supported by the core facility “Molecule Archive” of the German Research Foundation (10.13039/501100001659Deutsche Forschungsgemeinschaft, DFG project number: 284178167). J.W. and G.D. acknowledge funding by the 10.13039/501100001659Deutsche Forschungsgemeinschaft (DFG, German Research Foundation) – project number 331351713 – SFB 1324 (project A06 to G. Davidson). This work is in part supported by 10.13039/100000066National Institute of Environmental Health Sciences (NIEHS) training grant T32ES007060 (L-W. L.) and the American Cancer Society Mission Boost award (MBGI-25-1520234-01-MBG) (S. K. K.).

## Author contributions

Conceptualization, A.C.B.C., J.W., S.T., B.B., L.B., and W.B. data curation and analysis, K.K, C.B, S.M., M.R., S.S., I.S., H.T.W., J.M., M.G., J.S., Z.W., M.P., R.H., C.W.G., L-W.L., and D. M. resources, S.B., L.B., S.K.K., S.A., N. J., and S.B. supervision, A.C.B.C., C.B., S.K.K., P.B., S.T., B.B., L.B., W.B., and G.D. funding acquisition, N.J., S.B. B.B. L.B., and G.D. writing – original draft, A.C.B.C., S.M., L.B., and B.B. Writing-review and editing, all coauthors.

## Declaration of interests

J.S., M.P., S.B., N.J., S.B., and A.C.B.C. report patent application on the imidazopyridine compounds (pending). The other authors declare no competing interests.

## STAR★Methods

### Key resources table

**Table:** 

REAGENT or RESOURCE	SOURCE	IDENTIFIER
**Antibodies**		

Anti-Estrogen Receptor (F-10)	Santa Cruz	Cat# sc-8002; RRID: AB_627558
Anti-Estrogen Receptor (D547)	Santa Cruz	Cat# sc-53490; RRID: AB_629460
Anti-PCNA (PC10)	Santa Cruz	Cat# sc-56; RRID: AB_628110
Anti-Aryl Hydrocarbon Receptor (D5S6H)	Cell Signaling	Cat# 83200; RRID: AB_2800011
Goat anti-mouse IgG/HRP	DAKO	Cat# P0447; RRID: AB_2617137
Goat-*anti*-Rat-FITC	Novus Biologicals	Cat# NB7124
Anti-β-actin	Santa Cruz	Cat# sc-47778; RRID: AB_2714189
Anti-Vinculin	Santa Cruz	Cat# sc-73614; RRID: AB_2941767
Anti-Estrogen Receptor	Thermo Fisher	Cat# MA5-13304; RRID: AB_11002193

**Bacterial strain**		

NEB® 5-alpha Competent E. coli (High Efficiency)	New England Biolabs	Cat# C2987H

**Chemicals and recombinant proteins**		

(Dulbecco’s Modified Eagle’s Medium) DMEM DMEM without phenol red	Gibco Gibco	Cat# 41966-029 Cat# 21063-029
Roswell Park Memorial Institute (RPMI) 1640 RPMI 1640 Without phenol red	Gibco Gibco	Cat# 11875-085 Cat# 11835030
Fetal Bovine Serum (FBS)	Gibco	Cat# 10270-106
17 β-Estradiol	Sigma Aldrich	Cat# E8875
Fulvestrant	Sigma Aldrich	Cat# I4409
Indirubin	Sigma Aldrich	Cat# SML0280
Dioxin	Campro Scientific	Cat# ED-901
CH-223191	MedChemExpress	Cat# HY12684
Penicillin- Streptomycin	Gibco	Cat# 15140122
L-glutamine	Gibco	Cat# 25030081
Insulin-Transferrin-Selenium (ITS)	Thermo Fisher	Cat# 41400045
Human epidermal growth factor (EGF)	Miltenyi Biotech	Cat#130-093-825
Basic fibroblast growth factor	Miltenyi Biotech	Cat#130-093-840
Hydrocortisone	Sigma Aldrich	Cat# H0135
Choleratoxin	Sigma Aldrich	Cat# C8052
Lipofectamine 3000	Thermo Fisher	Cat# L3000001
Dimethyl sulfoxide	Carl Roth	Cat# A994.2
Passive lysis buffer	Promega	Cat# E1941
Collagenase	Merck Life Science	Cat# C9407
Cultrex Basement Membrane Extract (BME), Type 2	R&D Systems	Cat# 3533-005-02
Cell recovery	Corning	Cat# 354253
Dulbeccoś phosphate buffered saline	Gibco	Cat# 14190094
1,4-Dithiothreitol	Carl Roth	Cat# 6908.2
Adenosine-5′-triphosphate-disodium salt	Carl Roth	Cat# HN35.2
D-Luciferin Firefly	Biosynth/Carbosynth	Cat# L-8200
EGTA	Carl Roth	Cat# 3054.1
Potassium dihydrogen phosphate	Carl Roth	Cat# P018.1
di-Potassium hydrogen phosphate	Carl Roth	Cat# P749.1
Sodium chloride	Fischer Scientific UK	Cat# 11984051
Potassium chloride	AppliChem	Cat# 131494
Potassium dihydrogen phosphate	AppliChem	Cat# 141509
Sodium monohydrogen phosphate dihydrate	Fagron	Cat# 181620
Furosemid	Fagron	Cat# 700233
Phenytoin	Fagron	Cat# 700377
Caffeine	Sigma Aldrich	Cat# C0750
Imipramine hydrochloride	TCI Japan	Cat# I0971
Ethylenediamine tetraacetic acid disodium salt dihydrate	Carl Roth	Cat# 8043.1
Coelenterazine	Biosynth/Carbosynth	Cat# C-7002
Acetonitrile (LC-MS Chromasolv, Honeywell Riedel-de Haen)	Fisher Scientific	Cat # 15684740
Formic acid	Fisher Chemicals	Cat# A117-50
Phenyl methylsulfonyl fluoride (PMSF)	Sigma Aldrich	Cat# P-7625
IGEPAL	Sigma Aldrich	Cat# 18896
Alexa 488-GST	Thermo Fisher	Cat# A-11131
ERα LBD-GST	Thermo Fisher	Cat# PV4543
Crystal Violet	SERVA	Cat# 27335.01
Trypan blue	Thermo Fisher	Cat# 15250-061
Matrigel	Corning	Cat# 354248
Complete^TM^ Protease Inhibitor Cocktail	Roche	Cat# 04693124001
Sodium Orthovanadate (Na_3_VO_4_)	Merck	Cat# 567540
Sodium fluoride (NaF)	Merck	Cat# 201154

**Critical commercial assays**		

Venor®GeM Classic Mycoplasma Detection Kit	Minerva Biolabs	Cat# 11-1250
InnuPrep RNA Mini Kit 2.	Analytik Jena	Cat# 845-KS-2040250
M-MLV Reverse Transcriptase	Promega	Cat# M1701
SYBR Green GoTaq PCR mix	Promega	Cat# A6002
CellTiter-Glo® 3D Cell Viability Assay	Promega	Cat# G7570
PolarScreen™ Estrogen Receptor-Alpha Competitor Assay, Red	Thermo Fisher	Cat# A15884
CyQUANT^TM^ NF Cell Proliferation Assay Kit	Thermo Fisher	Cat# C35006

**Deposited data**		

The RNA-seq data deposited at the GEO repository	Pan et al.^17^	GSE218556

**Experimental models: Cell lines**		

MCF-7 luc	Harrod et al.^21^	N/A
MCF-7 luc D538G	Harrod et al.^21^	N/A
MCF-7 luc Y537S	Harrod et al.^21^	N/A
HeLa	ATCC	Cat# CCL-2; RRID: CVCL_0030
MCF-7	ATCC	RRID: CVCL_0031
T47D	ATCC	RRID: CVCL_0553
CTC-ITB-01	Koch et al.^18^	N/A

**Oligonucleotides**		

Human PGR For	5′-CTTAATCAACTAGGCGAGAG-3′	Metabion
Human PGR Rev	5′-AAGCTCATCCAAGAATACTG-3′	Metabion
Human GREB For	5′- GAGTGACAATGAGGAAGAG-3′	Metabion
Human GREB Rev	5′-CTCGTTGGAAATGGAGACAA-3′	Metabion
Human PDZK1 For	5′-GCAGGCTCAGAACAGAAAGG-3′	Metabion
Human PDZK1 Rev	5′- TCCAGGGTTTCCACAGACTC-3′	Metabion
Human TFF1 For	5′-CAATGGCCACCATGGAGAAC-3′	Metabion
Human TFF1 Rev	5′-AACGGTGTCGTCGAAACAGC-3′	Metabion
Human Cyp1A1 For	5′ TCAGCTCAGTACCTCAGCCA 3'	Metabion
Human Cyp1A1 Rev	5′ CATGGCCCTGGTGGATTCTT 3'	Metabion
Human CYP1B1 For	5′CCACTATCACTGACATCTTC 3′	Metabion
Human CYP1B1 Rev	5′ ACGACCTGATCCAATTCT 3'	Metabionn
Human ALDH3A1 For	5′ TGACTACATCCTCTGTGA 3'	Metabion
Human ALDH3A1 Rev	5′ GCACTAATGATTCTTCCATAG 3'	Metabion
Human β-actin For	5′ CTCCTGAGCGCAAGTACTCC 3′	Eurofins MWG Operon
Human β-actin Rev	5′GTCACCTTCACCGTTCCAGT 3′	Eurofins MWG Operon etabion
AHR protospacer 805 For	5′ CCTACGCCAGTCGCAAGCGG 3′	Metabion
AHR protospacer 805 Rev	5′ CCGCTTGCGACTGGCGTAGG 3′	Metabion
AHR protospacer 708 For	5′ CCAGCCTACACCGGGTTCCG 3′	Metabion
AHR protospacer 708 Rev	5′CGGAACCCGGTGTAGGCTGG 3′	Metabion

**Recombinant DNA**		

XRE(TnGCGTG)_3_-tata-luciferase-Luc-hygromycin plasmid	N/A	Gruszczyk et al.^29^
ERE-βGlobin-luciferase-Luc-neomycin	N/A	Delfosse et al.^59^
pSG5-hERα-puromycin	N/A	Delfosse et al.^59^
pSpCas9(BB)-2A-GFP (PX458)	Addgene	Cat# 48138

**Software and algorithms**		

ImageJ	N/A	https://imagej.nih.gov/ij/download.html
ColonyArea	Guzmán et al.^20^	https://imagej.net/plugins/colonyarea
GraphPad Prism8	GraphPad	https://www.graphpad.com/
R (version 4.5.2)	R programming language (R Foundation)	https://www.r-project.org
tidyverse (v2.0.0)	CRAN	https://cran.r-project.org/package=tidyverse
EBImage (v4.52.0)	Bioconductor	https://bioconductor.org/packages/EBImage
imager (v1.0.8)	CRAN	https://cran.r-project.org/package=imager
SoftMax Pro 7	Molecular Devices	N/A
GSEA Software v4.1.0	Mootha et al.^60^; Subramanian et al.^61^	http://www.gsea-msigdb.org/gsea/login.jsp;jsessionid=3B86CA472E2D1D71844F8EC8F4872140
Image Lab	Bio-Rad	https://www.bio-rad.com/de-de/product/image-lab-software?ID=KRE6P5E8Z
Tycho NT.6 instrument	NanoTemper Technologies	https://www.xtal.iqf.csic.es/TBIO/laboratory/Tycho-NT.6-User-Manual_V08.pdf
Glide (Schrödinger Release 2025-1, version 10.6)	Schrödinger, LLC	https://www.schrodinger.com/products/glide

### Experimental model and study participant details

#### Cell lines

All cell lines except HAhLH and HELN hERα cells were obtained from the American Type Culture Collection (ATCC): MCF-7 (RRID:CVCL_0031), T47D cells (RRID: CVCL_0553), HeLa (CCL-2, RRID:CVCL_0030). CRISPR ER knock-in cell lines (MCF-7 luc, MCF-7 luc D538G and MCF-7 luc Y537S) have been reported previously.^21^ The identities of the cells were confirmed by short tandem repeat profiling (BioSynthesis, Lewisville, TX and DSMZ Braunschweig, Germany). The cell lines were routinely confirmed to be *mycoplasma*-free, using the VenorGeM Classic Mycoplasma Detection Kit for conventional PCR (Minerva Biolabs, 11–1250). MCF-7 was cultured in DMEM (Gibco, Thermo Fisher), supplemented with 10% fetal bovine serum (FBS) (Gibco, Thermo Fisher), 1% penicillin/streptomycin (Gibco, Thermo Fisher). T47D, MCF-7 luc, MCF-7 luc D538G and MCF-7 luc Y537S cells were cultured in RPMI 1640 (Gibco, Thermo Fisher), medium supplemented with 10% fetal bovine serum (FBS), 1% penicillin/streptomycin. The T47D culture medium was additionally supplemented with 0.6 μg/mL insulin. HAhLH cell line was cultured in DMEM-F12 supplemented with 5% fetal bovine serum (FBS), 1% penicillin/streptomycin, 0.25 mg/ml hygromycin. HELN hERα cells were cultured in DMEM-F12 supplemented with 5% FBS, 1% penicillin/streptomycin, 0.5 μg/ml puromycin and 1 mg/mL geneticin. CTC-ITB-01 cells have previously been described^18^ and were cultured in RPMI 1640 medium supplemented with 10% fetal bovine serum (FBS), 1% penicillin/streptomycin, 1% Insulin-Transferrin-Selenium (Gibco, Thermo Fisher), 50 ng/mL Epidermal growth factor (Miltenyi Biotech), 10 ng/mL human basic fibroblast growth factor (Miltenyi Biotech), 100 ng/mL hydrocortisone (Sigma), 200 ng/mL choleratoxin (Sigma). All cells were maintained at 37 °C in an incubator with 5% CO_2_ and 90% humidity. Unless otherwise stated, hormone treatment was carried out in cells that have been previously hormone starved. This requires culturing the cells for 72 h in phenol red-free RPMI 1640 or DMEM media, supplemented with 3% charcoal-stripped fetal calf serum (CCS).

#### Generation of patient derived xenograft (PDX)-derived organoids (PDxO)

##### Ethical statement

Human specimens were collected from breast cancer patients undergoing surgery at the National Cancer Institute, Aviano, after signing an informed consent form. Permission for the studies was granted by the Institutional Review Board of CRO Aviano (IRB-06-2017) in agreement with all relevant ethical regulations, including the Declaration of Helsinki.

##### Procedure

Organoids were generated from patients’ samples and from patient-derived xenograft (PDX) tumors, as previously described.^25^ PDX were derived from BCRO#28, an ER^+^ breast cancer sample collected from a treatment naive 57 years-old patient. PDO#268 was derived from BCRO#268, an ER^+^ breast cancer sample collected from treatment naive 43-years-old patient. PDO#480 was derived from BCRO#480, an ER^+^ breast cancer sample collected from a 39-years-old patient subjected to chemotherapy in neo-adjuvant setting. Briefly, tumors freshly explanted from mice were mechanically shredded, digested with collagenase (Merck) and filtered through a 100 μm strainer. Digested material (isolated cells and small cell clusters) was centrifuged and embedded in 3D-matrix drops (reduced growth factor Basement Membrane Extract, BME, R&D systems), then overlaid with breast cancer organoid medium.^25^ For 3D survival curves under treatment, organoids were embedded in BME drops and, after 24 - 48 h, drugs were added. Fresh drug-containing medium was replaced on day 4. On day 7 of treatment, CellTiter-Glo 3D Cell Viability Assay (Promega) was added and luminescent signal from viable organoids was recorded on Infinite M1000 Pro instrument (Tecan).

### Method details

#### Reporter cell line assay

To characterize the chemical induced AHR activity, we used the already established HAHLH reporter cell lines obtained by stably expression of HeLa cells with the dioxin-responsive gene XRE(TnGCGTG)3-tata-luciferase-Luc-hygromycin plasmid.^29^ The reporter HELN hERα cell line was obtained by transfecting human HeLa cells with the ERE-responsive gene ERE-βGlobin-luciferase-Luc-neomycin and the pSG5-hERα-puromycin plasmids as previously described.^59^ To determine the action of the chemical on AHR and hERα activity, cells were seeded at a density of 50,000 cells per well in 96-well white opaque tissue culture plates (Greiner CellStar) in 150 μL of Dulbecco’s Modified Eagle Medium: Nutrient Mixture F-12 (DMEM/F- 12) without phenol red and 1 g/L glucose and supplemented with 5% stripped fetal bovine serum, 100 units/mL of penicillin, 100 μg/mL of streptomycin (test medium). Twenty-four hours later, chemicals to be tested were added at 4x concentration in culture medium, and cells were incubated at 37 °C for 8 h (HAhLH cells) or 24 h (HELN hERα cells). At the end of the incubation period, culture medium was replaced with test medium containing 0.3 mM luciferin and luciferase activity was measured for 2 s in intact living cells using a MicroBeta Wallac luminometer (PerkinElmer). Tests were performed in quadruplicate at 4x concentration in the same medium in at least 4 independent experiments. Results were expressed as % of the maximal luciferase activity. Maximal luciferase activity (100%) was obtained in the presence of 10 nM dioxin (HAhLH cells) or E_2_ (HELN hERα cells). Effective concentration (ECs) for a given compound, EC_50_ is defined as the concentration inducing 50% of its maximal effect. The EC_50_ values were calculated including the adjustment for the basal activity of the cell line.

#### CRISPR/Cas9 knock out

The human AHR locus was targeted within exon 1 to introduce a frameshift. Using the CRISPOR online tool,^62^ two 20 bp protospacer (805 5′-CCTACGCCAGTCGCAAGCGG-3′, 708 5′-CCAGCCTACACCGGGTTCCG-3′) followed at the 3′ end by an NGG PAM (protospacer adjacent motif) upstream and downstream of the AHR start codon were identified. DNA oligonucleotides were designed and annealed to generate short double-stranded DNA fragments containing the 20 bp protospacer and an additional guanine nucleotide at the 5′end was added to increase targeting efficiency^63^ and 4 bp overhangs suitable for ligation into a Bbs I restriction site. This short double-stranded DNA fragments were ligated into the Bbs I restriction site of pSpCas9(BB)-2A-GFP (PX458) vector, a gift from Feng Zhang (#48138, Addgene) and transformed in NEB 5-alpha Competent *E. coli* bacteria for plasmid preparation. To knock out AHR, T47D or MCF-7 cells cultured in 6-well plates were transfected with 2.5 μg μg pSpCas9(805)-2A-GFP to induce a frameshift or with 1.25 μg pSpCas9(805)-2A-GFP and 1.25 μg pSpCas9(708)-2A-GFP to delete ATG using Lipofectamine 3000 (Thermo Fisher Scientific) according to the manufacturer’s 1-step protocol. Forty-eight hours after transfection, GFP-positive cells were sorted using Fluorescence-Activated Cell Sorting (FACS), and single cell clones were expanded for further characterization. After SDS-PAGE and Western blot screening of the FACS sorted clonal cell lines, genomic DNA from promising candidates was PCR amplified at the region of interest (For. 5′- CCAGGCAGCTCACCTGTAC -3'; Rev. 5′- CTCTAACCTAACCCATGCGGATATG – 3′) and sent for sequencing (Microsynth AG, Balgach, Switzerland).

#### Western blotting

To determine the relative expression of proteins by Western blotting, cells were first washed with ice-cold phosphate buffered saline (PBS). Subsequently, they were lysed in ice-cold NP40 lysis buffer (1%NP-40, 50 mM Tris-HCl, pH 8.0, 150 mM NaCl, 5 mM Ethylenediaminetetraacetic acid [EDTA], 1 mM PMSF) on ice. DNA was degraded by sonification (five pulses at 50 amplitudes). Protein concentration was determined, and equal amounts of protein were separated on a 9% SDS-PAGE gel. The proteins were transferred onto nitrocellulose membrane by a standard Western blotting protocol and the membranes were incubated with the following antibodies: ERα (Santa Cruz Biotechnology, sc-8002, RRID: AB_627558) and PCNA (Santa Cruz Biotechnology, sc-56, RRID: AB_628110), followed by incubation with HRP labeled Goat anti-mouse Immunoglobulins (DAKO, P0447, RRID: AB_2617137).

To obtain protein extracts from PDOs, organoids were incubated for 2 h on ice in a cell recovery solution (Corning, 354253) to degrade the Matrigel. Then, after 5 min centrifugation at 1,500 rpm at 4°C, pellet containing PDOs was resuspended in an appropriate volume of cold RIPA lysis buffer (NaCl 150 mM, 50 mM Tris HCl pH 8, 1% IGEPAL, 0.5% sodium-deoxycholate, 0.1% SDS in deionized water), supplemented with a protease inhibitor cocktail (CompleteTM, Roche), 1 mM Na_3_VO_4_, 100 mM NaF and 1 mM DTT (Merck). Organoids were then incubated for 30 min on ice and centrifuged at maximum speed for 30 min at 4°C to obtain protein lysates. Immunoblotting was carried out using standard protocols, with 7 μg of input protein *per* lane, and with the following antibodies: vinculin (Santa Cruz; sc-73614; 1:1000); ERα (Thermo Fisher; MA5-13304; 1:200).

#### Fluorescence polarization assay

The PolarScreen Estrogen Receptor-Alpha Competitor Assay, Red (ThermoFisher, A15884) was used to determine if the X compounds bind to ERα. The assay was performed according to the manufacturer’s protocol. In brief, 34 nM ERα full-length and 1.4 nM Fluormone EL Red were incubated for 4 h at room temperature together with known concentrations of estradiol, fulvestrant or the X compounds. Fluorescence polarization was measured in a Tecan F200 reader with 535 nm excitation and 595 nm emission interference filters.

#### Clonogenic assay

Cells were seeded at a density of 1–2×10^3^ cells/well in a 6-well plate and treated with increasing concentrations of the X compounds for 14–21 days. Medium and compounds were renewed every 7 days. The experiments were terminated by fixing the cells with methanol and the colonies formed were visualized after staining with 0.5% crystal violet (w/v) in 20% methanol (v/v). Plates were photographed and the area covered by the colonies was calculated using the ColonyArea Plugin for Fiji (RRID:SCR_003070).^20^

#### Cell proliferation assay

For the proliferation assay, indicated cell lines were plated in a 24-well plate format at 1x10^4^ cells/well. Cells were cultured for 5 days, and cell growth was determined by trypan blue exclusion using the direct cell count function on a Countess II (Life technologies).

#### Determination of LC50 values with CyQUANT

LC50 values were measured using the CyQUANT NF Cell Proliferation Assay Kit (Thermo Fisher). CTC-ITB-01 cells were plated at a density of 2x10^3^ cells/well in 96-well plates and treated the following day with serial dilutions of the test compounds. After incubation for the indicated time, cells were stained with CyQUANT dye according to the manufacturer’s protocol and incubated at 37°C for 30 min. Fluorescence was measured using a Tecan Infinite M Nano+ (excitation: 485 nm; emission: 530 nm). For the calculation of the LC_50_ the fluorescence was plotted against the Log_10_ of the inhibitor concentration in nM as x values and the LC_50_ values were calculated with 95% confidence intervals (*n* = 4).

#### Bulk RNA sequencing and quantitative RT-PCR experiments

Bulk RNA sequencing was previously performed with MCF-7 and T47D cells treated with 1 μM X15695 in the presence and absence of 10 nM 17-β-estradiol (E_2_). The sequencing data was deposited at the GEO repository under accession number GSE218556. The bulk RNA sequencing data were analyzed by R (v4.5.1) with related R packages. The differentially expressed genes (DEGs) between two conditions and groups (three biological replicate per condition) were analyzed by R package limma (v3.64.3).^64^ *p*-values were adjusted by using the Benjamini and Hochberg’s approach for controlling the False Discovery Rate (FDR). DEGs with a fold change of Log2(CPM) ≥ 1.0 or ≤ −1.0 and an adj. *p* value ≤0.05 were considered as significantly regulated. To visualize the fold change of DEGs that were enriched in hallmark pathway, heatmaps were generated by R package pheatmap (v1.0.13).^65^ Gene set enrichment analysis (GSEA) for hallmark pathway were performed by the R package clusterProfiler (v4.17.0).^66^ Selected targets of DEGs were verify by quantitative RT-PCR experiments with the SYBR Green GoTaq PCR mix (Promega, A6002).

#### Thermal stability measurements (nano-DSF)

Thermal denaturation experiments of AHR complexes in the presence of compounds were performed using a Tycho NT.6 instrument (NanoTemper Technologies). Protein samples were prepared as previously described^67^ and subsequently diluted in the buffer containing 20 mM Bis-Tris–HCl pH 7.0, 50 mM NaCl, 10 mM KCl, 10 mM MgCl_2_, 20 mM Na_2_MoO_4_ and 2 mM β-mercaptoethanol to a final protein concentration of 2.5 μM. Compound stock solutions were prepared at 20 mM in DMSO and added to the protein samples to yield a final concentration of 12.5 μM. For control measurements, DMSO was added to a final concentration of 5% (v/v). Following ligand or DMSO addition, the samples were gently mixed and incubated at room temperature for 10 min to allow equilibration. Thermal denaturation profiles were acquired over a temperature range of 35°C–95°C, with a heating rate of 1 °C per min. Fluorescence was monitored at emission wavelengths of 330 nm and 350 nm, and the data were analyzed as the first derivative of the fluorescence ratio (F_330_/F_350_) as a function of temperature. All experiments were performed in triplicate, and data were analyzed using the Tycho NT.6 software (NanoTemper Technologies) according to the manufacturer’s recommendations.

#### Nuclear receptor activity profiling (NAPing)

Preparation of assay mixes with ERα recombinant ligand binding domain (LBD) and full-length ERα in MCF-7 extracts and measurement of coregulator recruitment on the NAPing platform (PML, Oss, The Netherlands) was performed as described previously.^68^^,^^69^ In short, samples with ERα LBD-GST (Invitrogen #PV4543) or full-length protein were treated with −8.5 logM (∼EC_50_) of estradiol (E_2_) and subsequently incubated with 1000-fold excess (−5.5 logM) of each test compound and incubated for 30 min at room temperature on the NAPing platform with 101 coregulator-derived binding NR-binding motifs. ERα binding was detected using Alexa 488-GST (Thermo Fisher #A-11131, for LBD) or ERα/Goat-*anti*-Rat-FITC (Santa Cruz sc-53490/Novus Bio NB7124) antibodies. Unbound ERα and antibodies were removed by washing. Residual ERα binding to each coregulator motifs was recorded on tiff images and quantified using an R-based analysis software package (PML, Oss, The Netherlands).

#### Determination of thermodynamic solubility

Thermodynamic solubility was determined using the classical shake-flask method. The solid compound (4 mg) was suspended in 1 mL HEPES buffer (0.1 M, pH 7.4) and constantly stirred at 21 °C for 24 h. Thereafter, the suspension was centrifuged at 13,000 rpm for 5 min (Centrifuge Mikro20, Andreas Hettich GmbH) and the clear supernatant was transferred to an HPLC vial (flat bottom, borosilicate glass 5.1, 1.5 mL, ND9 (Cat. No. 548-0028AP, VWR International) in combination with 9 mm natural rubber/TEF screw caps (Cat. No. 11722438, Fisher Scientific) and analyzed using an Agilent Single Quadrupole LC/MSD iQ Mass-System equipped with a Kinetex 2.6 μm XB-C18 100 Å LC column (100 × 4.6 mm). The Absorption (λ = 254 nm) was measured and the Area Under the Curve evaluated (AUC). The mobile phase used consisted of solvent A: Ultrapure water obtained from a Sartorius Arium Pro system containing 0.1% formic acid (Cat. No. A117-50, Fisher Chemical) and solvent B: Acetonitrile (LC-MS Chromasolv, Cat. No. 15684740, Honeywell Riedel-de Haen) containing 0.1% formic acid. The injection volume for all analyses was 50 μL and the flow rate was kept at 1 mL/min during the analysis. A mobile phase gradient method was applied as a method with 5% solvent B to 99% solvent B in 5 min, followed by 3 min of elution at 99% solvent B.

Calibration curve was generated from a serial dilution of the respective analyte in DMSO at 2.5, 1.25, 0.625, 0.313, 0.156, 0.078, 0.039 mM starting from a 10 mM DMSO stock solution. Subsequently, 2 μL of each dilution was added to 198 μL of a buffer (HEPES, 0.1 M, pH 7.4)/acetonitrile mixture in a 3:2 ratio yielding final dilutions of 25, 12.5, 6.25, 3.125, 1.562, 0.781, 0.391 μM. These were then subjected to HPLC analysis as described above.

Using the results from the linear regression of the internal calibration measurement points (Figure S5) and the mean AUC values from the saturated aqueous solutions, the concentration C of the analytes in the saturated aqueous solution, expressed in μM, were calculated using Equation 2:

(Equation 1) AUC=Slope·C+YIntercept

(Equation 2) $$C=\frac{AUC-YIntercept}{Slope}$$

#### Compound stability measurements in aqueous environment

The stability of the compounds was evaluated at a concentration of 12.5 μM in a 3:2 mixture of buffer (HEPES, 0.1 M, pH 7.4) and acetonitrile, with 1% DMSO. Acetonitrile was added because the concentration of 12.5 μM exceeded the thermodynamic solubility of the compounds in the buffer. This addition thereby prevented the formation of precipitates and a gradual decrease in compound concentration during the measurement. The resulting solution was stirred at 21 °C in a sealed vial (crimp neck vials, 5 mL, clear (Cat. No. 548-0611A) in combination with natural rubber septa (548-0565A) and aluminum crimp caps (548-3278A), VWR International) for 5 days. Samples of 100 μL were collected after 0, 24, 48, 72, 96, and 120 h and analyzed using HPLC as described above for the thermodynamic solubility determination. The AUC measured was subsequently plotted as a function of the incubation time (Figure S3). No signs of decomposition could be observed over the given time.

#### Parallel artificial membrane permeability assay

Passive membrane permeability was determined using Corning BioCoat Pre-coated PAMPA plates (Corning, Cat.# 353015). The plates were stored at – 20 °C until ready for use. Prior to use, the plate was prewarmed at room temperature for 30 min. Donor- and acceptor solutions were prepared as 12 mM phosphate buffered saline with a pH of 6.5 (donor) respectively 7.4 (acceptor). The pH was measured using a pH meter 766 (Knick, Germany) that was calibrated prior to use. All test compounds were prepared as 5 mM stock solutions in DMSO. Test solutions were prepared for the reference compounds furosemide, phenytoin, imipramine hydrochloride and caffeine via dilution with donor media to a starting concentration of 200 μM. X15695 was diluted with donor solution and additional 10% ethanol as solubility-enhancing additive. Then, 300 μL of each donor solution were filled into the donor (bottom) wells of the PAMPA plate. 200 μL of the acceptor medium were loaded into each well of the pre-coated receiver plate (top). The filter plate was placed on top of the donor plate and the whole PAMPA plate was incubated at 25 °C for 5 h without stirring. Thereafter, the donor and acceptor plate were separated and 100 μL of each well were transferred into solvent resistant 96-well plates (Eppendorf, Cat.# 0030 601.904). The compound concentrations in the donor- and acceptor wells were then determined using a Jasco 2 HPLC system equipped with a ReproSil-Pur 120 ODS-3 column (5 μm, 50 × 2 mm) and UV detection. Furosemide was measured at λ = 238 nm, Imipramine and Phenytoin at λ = 220 nm, Caffeine at λ = 275 nm and X15695 at λ = 254 nm. The mobile phase consisted of solvent A: Double distilled water containing 0.1% trifluoroacetic acid (Cat. No. T/3258/PB05, Fisher Scientific) and solvent B: Acetonitrile (Cat. No. A/0627/15, Fisher Scientific) containing 0.1% TFA. The injection volume was 40 μL and the flow rate 1 mL/min. A mobile phase gradient was applied with 1% solvent B to 99% solvent B in 3.5 min, followed by 1 min of elution at 99% solvent B. For determination of the compound concentrations in the donor- and acceptor solutions, calibration curves of all reference drugs were prepared in double distilled water containing 4% DMSO. For X15695, two calibration curves were prepared in the presence (donor well) and absence (acceptor well) of 10% ethanol. (Figure S6). The experiments were performed in triplicates.

The apparent permeability (P_app_) was determined using Equation 3

(Equation 3) $$P_{app}=\frac{-\ln\left(1-\frac{c_{A}\left(t\right)}{c_{equilibrium}}\right)}{A\times \left(\frac{1}{V_{D}}+\frac{1}{V_{A}}\right)\times t}$$

$$c_{equilibrium}=\frac{c_{D}\left(t\right)\times V_{D}+c_{A}\left(t\right)\times V_{A}}{V_{D}+V_{A}}$$

Where c_A_(t) is the compound concentration in the acceptor well in μmol⋅l^−1^, c_D_(t) is the compound concentration in the donor well in mol⋅l^−1^, V_A_ and V_D_ is the volume of the acceptor respectively donor solution per well in mL, A is the membrane area in cm^2^ and t the incubation time in seconds (Table S3).

### Quantification and statistical analysis

Experiments were performed with three or more replicates. Differences between two groups were analyzed by Student’s *t* test and multiple comparisons were determined by one-way ANOVA. For experiments where two factors (e.g., dose and time) were investigated, data were analyzed by two-way ANOVA followed by the appropriate post-hoc test. Data were expressed as means ± SEM, and *p* < 0.05 was considered significant. Statistical significance was set at ∗*p* < 0.05; ∗∗< 0.01; ∗∗∗< 0.001; ∗∗∗∗< 0.0001. All analyses were performed using Microsoft Excel 2010 or GraphPad Prism 8.3.1 software.
