# Supplementary material for: Distinct 2-phenylimidazo[1,2-a]pyridine derivatives that inhibit breast cancer cell proliferation identified as AHR ligands
Source: iScience. 2026 Apr 29;29(6):115936. doi: 10.1016/j.isci.2026.115936 (PMC13197777; doi:10.1016/j.isci.2026.115936)
Supplement: Document S1. Figures S1–S6, Tables S1–S3 and Data S1 [file mmc1.pdf]

iScience, Volume *29*

Supplemental information

Distinct 2-phenylimidazo[1,2-*a*]pyridine derivatives that inhibit breast cancer cell proliferation identified as AHR ligands

Katrin Koellisch, Christine Blattner, Stefano Motta, Janine Wesslowski, Melanie Rothley, Simone Büchel, Savannah Sirounian, Ilenia Segatto, Hanna T. Weber, Julia Müller, Marina Grimaldi, Jutta Stober, Zoe Wammetsberger, Mengwu Pan, René Houtman, Christoph W. Grathwol, Lo-Wei Lin, Laki Buluwela, Siva Kumar Kolluri, Dominik Mytzka, Simak Ali, Nicole Jung, Patrick Balaguer, Sonja Thaler, Barbara Belletti, Laura Bonati, William Bourguet, Stefan Bräse, Gary Davidson, and Andrew C.B. Cato

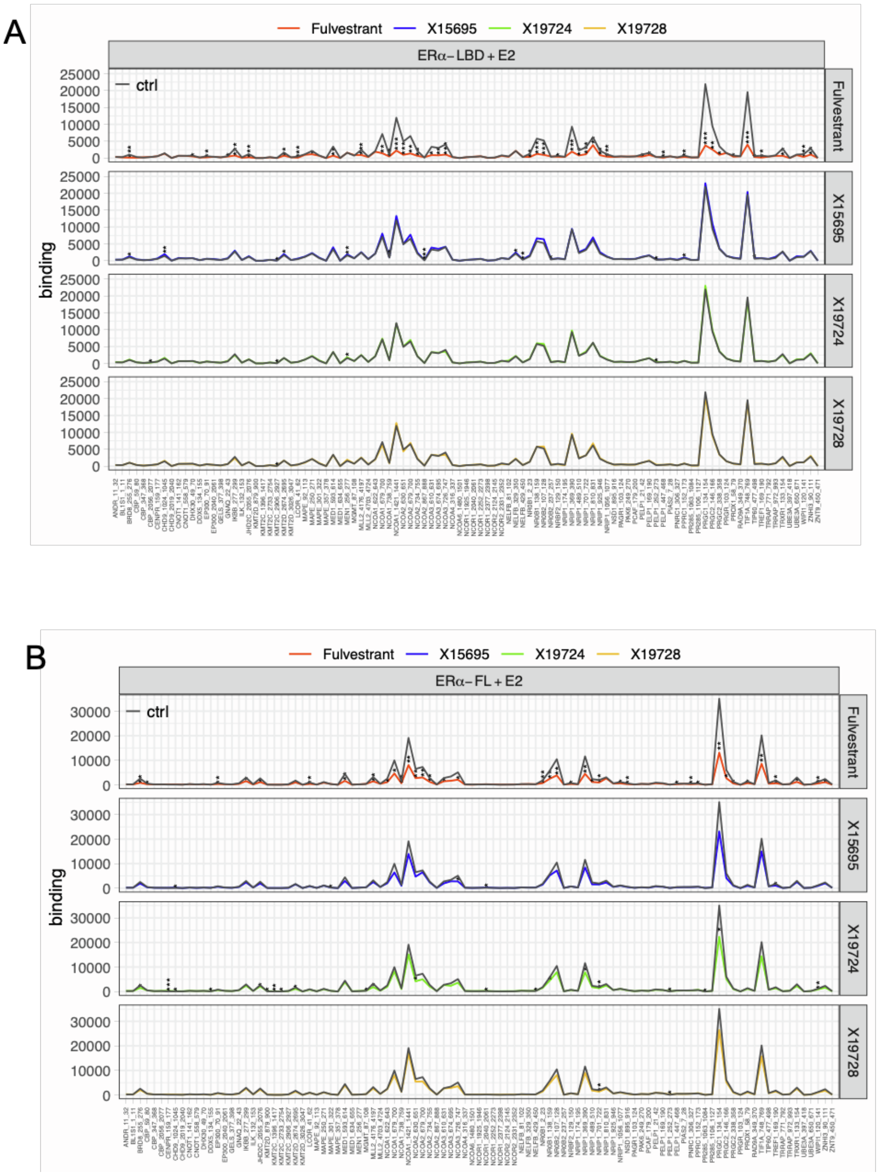

Figure S1. Imidazopyridines inhibit breast cancer cell proliferation independent of direct ERα binding. (Related to **Figure 3**). **(A)** Recombinant ERα LBD or **(B)** ERα FL in MCF-7 extracts was treated with E_2 EC_{50} (-8.5 logM) and subsequently incubated with 1000-fold excess (-5.5 logM) of each compound for 30 min. Each panel shows plots of the level of ER binding in the absence of compound (ctrl, E_2 only, black line) and presence of the test compounds (colored line, see legend) to each of 101 coregulator-derived NR-binding motifs on the NAPing platform. Binding is represented as mean of three technical replicates per indicated condition. Significance of test compound-induced modulation of ER control binding was assessed using Student's t-Test (*p<.05; **<0.01; ***<.0001).

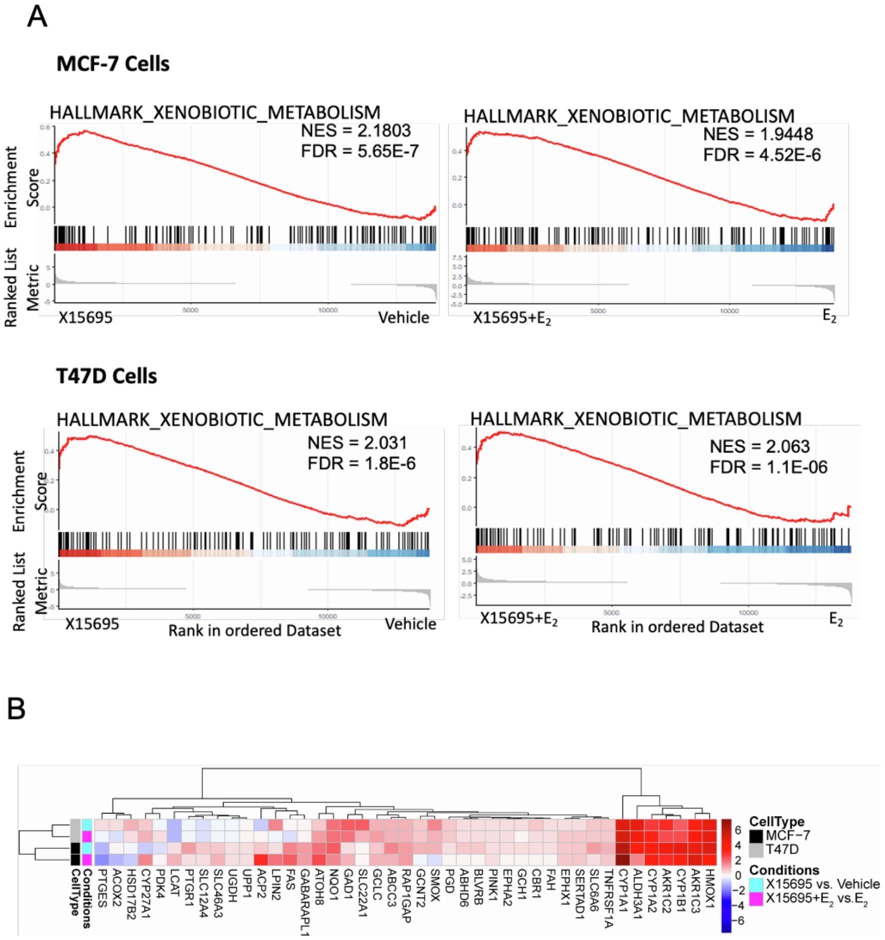

Figure S2. Hallmark gene analysis showing genes in the xenobiotic metabolism signaling pathways targeted by X15695. (Related to **Figure 4**). **(A)** Gene Set Enrichment Analysis (GSEA) plots of xenobiotic metabolism signaling pathway identified as one of the topmost gene sets in a comparison of X15695 vs vehicle and E_2 + X15695 vs E_2 in MCF-7 and T47D datasets. (**B)** Heatmaps of Log_2 fold-change in gene expression in the comparison of X15695 vs vehicle and E_2 + X15695 vs E_2 treatment of MCF-7 and T47D cells.

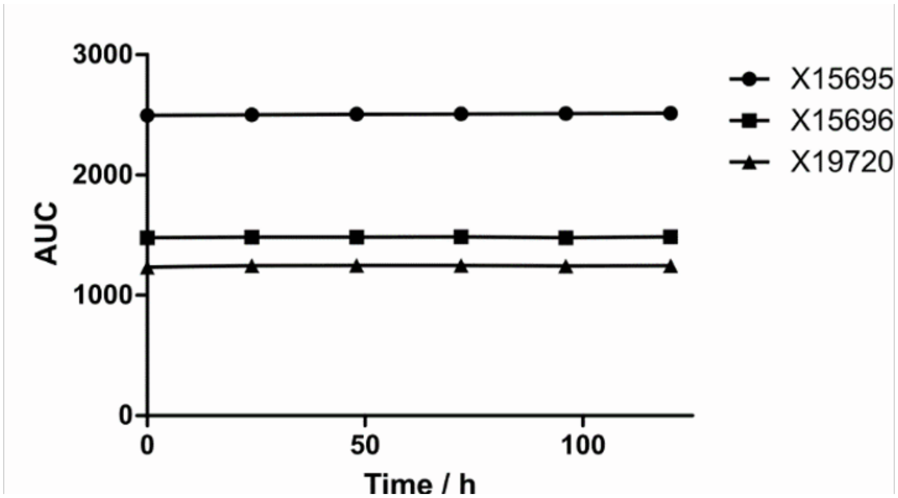

Figure S3. Stability of X15695, X15696 and X19720 in aqueous environments (Related to **STAR Methods**). Area under the curve (AUC) as a function of the incubation time in hours, measured via HPLC at λ = 254 nm.

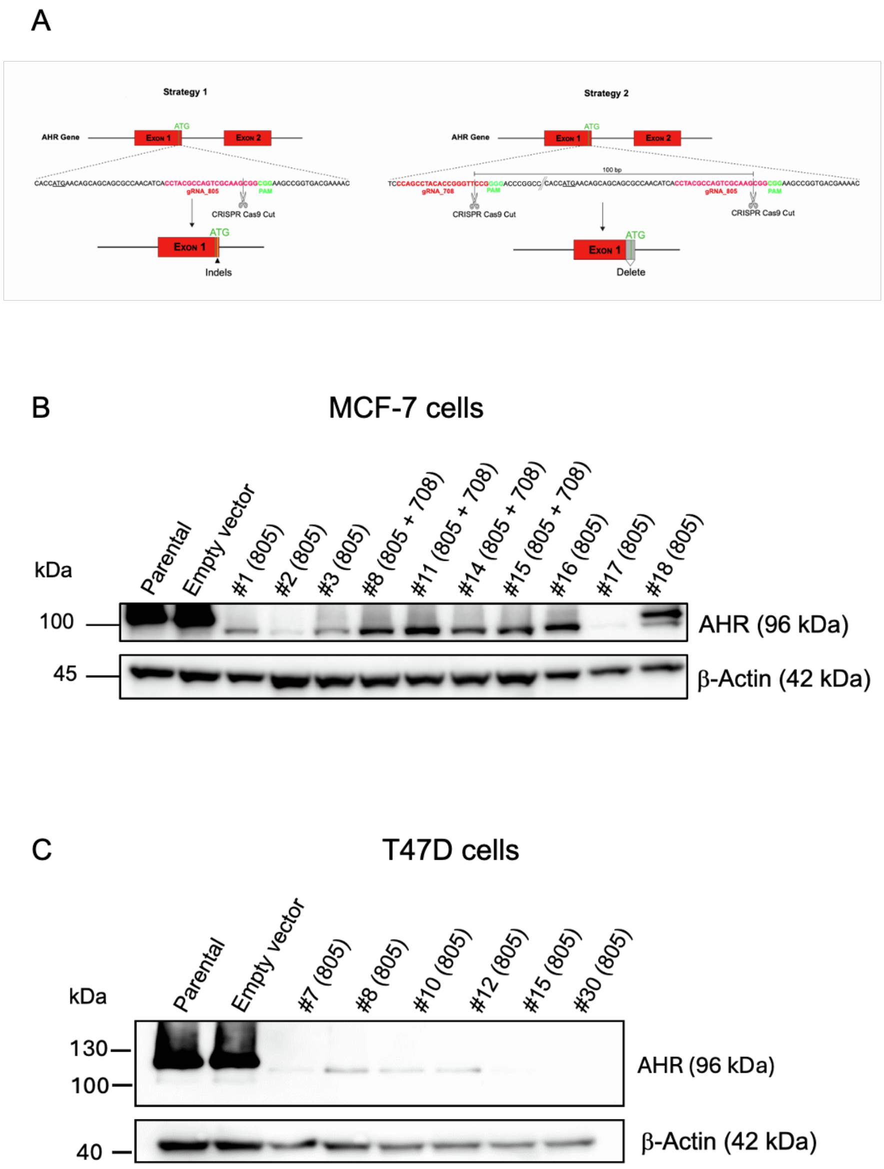

Figure S4. Generation of CRISPR/Cas9 knockout AHR cells. (Related to **Figure 6**). **(A)** Strategy for the generation of AHR knockout. In strategy 1, gRNA _805 was chosen to introduce a double-stranded DNA break immediately downstream of the *AHR* start codon so that repair by nonhomologous end joining will introduce indels to disrupt the protein reading frame. In strategy 2, gRNA_708 upstream and gRNA_805 downstream of the *AHR* start codon should cause two double-stranded DNA breaks in approx. 100 bp distance. Thus, repair by nonhomologous end joining will delete the start codon and block transcription. **(B and C)** Selection of AHR KO clones in MCF-7 and T47D cells. Western blot with 20 µg protein from parental, empty vector transfected cells and the AHR CRISPR Cas9 knockout clones in MCF-7 (B**)** and T47D (C) cells with different sgRNA. Anti-AHR and b-actin antibodies were used for the Western blots.

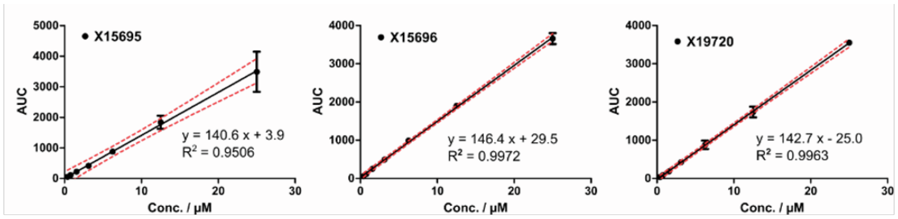

Figure S5. Determination of Thermodynamic Solubility. External calibration for thermodynamic solubility determination. Area under the curve (AUC) is presented as a function of the concentration of compounds X15695, X15696, and X19720 in µM measured via HPLC at λ = 254 nm. Linear regression of the measured values yields calibration curves (black line) for calculating the maximum thermodynamic solubility of the compounds. Error bars indicate the standard deviation of duplicate measurements. The 95% confidence interval is represented by the red dotted lines.

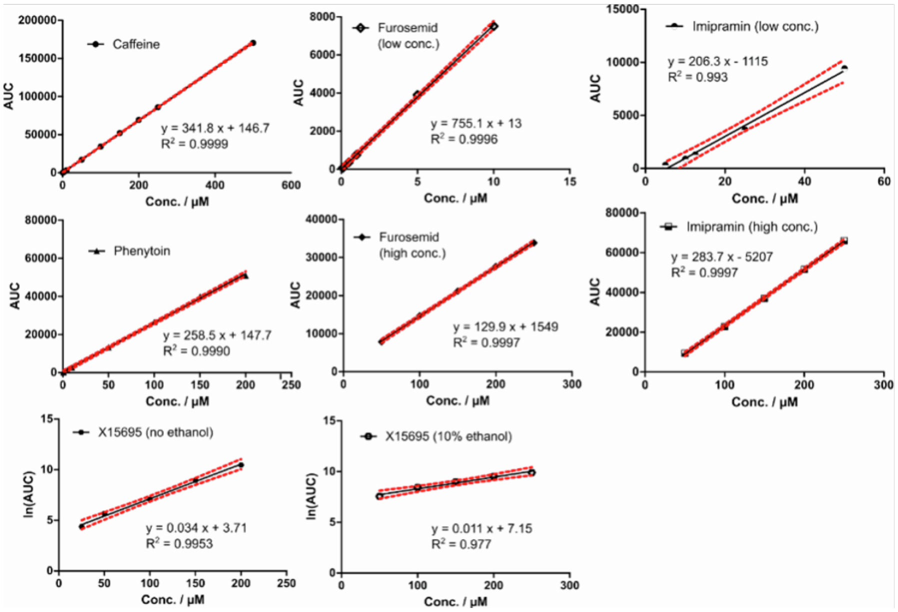

Figure S6. External calibration for membrane permeability determination. Area under the curve (AUC) as a function of the concentration of the reference drugs caffeine, imipramin, furosemide, phenytoin and compound X15695, measured via HPLC. Linear regression of the measured values yields calibration curves (black line) for calculating the concentration in the donor and acceptor well after incubation. The 95% confidence interval of the linear fit is represented by red dotted lines.

Table S1: Results on the thermodynamic solubility determination of analytes X15695, X15696, and X19720 (Related to **STAR Methods**)

	AUC (N = 1)	m/z found	AUC (N = 2)	m/z found	Mean AUC	Solubility / µM
**X15695**	532.606	314.8	391.862	314.7	423.653	2.99
**X15696**	783.832	297.1	771.367	297.0	777.600	5.11
**X19720**	164.348	315.2	172.102	315.0	168.225	1.35

Table S2: Results on the artificial membrane permeability (apparent permeability, P_{app}) of reference drugs and the compound X15695 (Related to **STAR Methods).**

Compound	Function	pH (donor)	Additives	Mean P_{app} / $cm \cdot s^{-1}$	SD
Furosemid	Reference, low P_{app}	6.5	DMSO (4%)	$0.132 \cdot 10^{-6}$	$0.16 \cdot 10^{-6}$
Imipramine	Reference, high P_{app}	6.5	DMSO (4%)	$3.42 \cdot 10^{-6}$	$0.36 \cdot 10^{-6}$
Phenytoin	Reference, moderate P_{app}	6.5	DMSO (4%)	$3.35 \cdot 10^{-6}$	$1.02 \cdot 10^{-6}$
Caffeine	Reference, high P_{app}	6.5	DMSO (4%)	$10.7 \cdot 10^{-6}$	$0.53 \cdot 10^{-6}$
X15695	Analyte	6.5	DMSO (4%)<br>Ethanol (10%)	$8.68 \cdot 10^{-6}$	$4.53 \cdot 10^{-6}$

Table S3: Results of concentration determinations of the reference drugs caffeine, imipramine, furosemide, phenytoin and X15695 in donor and acceptor wells via HPLC. AUC was converted into concentrations using the calibration curves from Figure S6 (Related to **STAR Methods).**

	Replica	Caffeine		Imipramine		Furosemid		Phenytoin		X15695	
		AUC	Conc. / µM	AUC	Conc. /µM	AUC	Conc / µM	AUC	Conc / µM	AUC	Conc / µM
Donor well	1	55085.9	160.76	53345.7	206.37	29834.8	217.83	24563.5	112.02	15026.5	214.69
	2	55322.1	161.45	50164.1	195.16	32055.4	234.94	25475.6	117.35	11362.6	190.41
	3	56615.4	165.23	49784.4	193.82	34926.2	257.04	26019.7	120.53	17114.1	225.99
Acceptor well	1	15151.4	43.91	2661.8	18.31	1374.5	1.80	6606.9	7.15	268.8	55.19
	2	14309.6	41.44	2209.6	16.11	394.8	0.51	7119.3	10.14	274.9	55.84
	3	15997.7	46.38	2916	19.54	67.4	0.07	7789.9	14.06	96.6	25.23

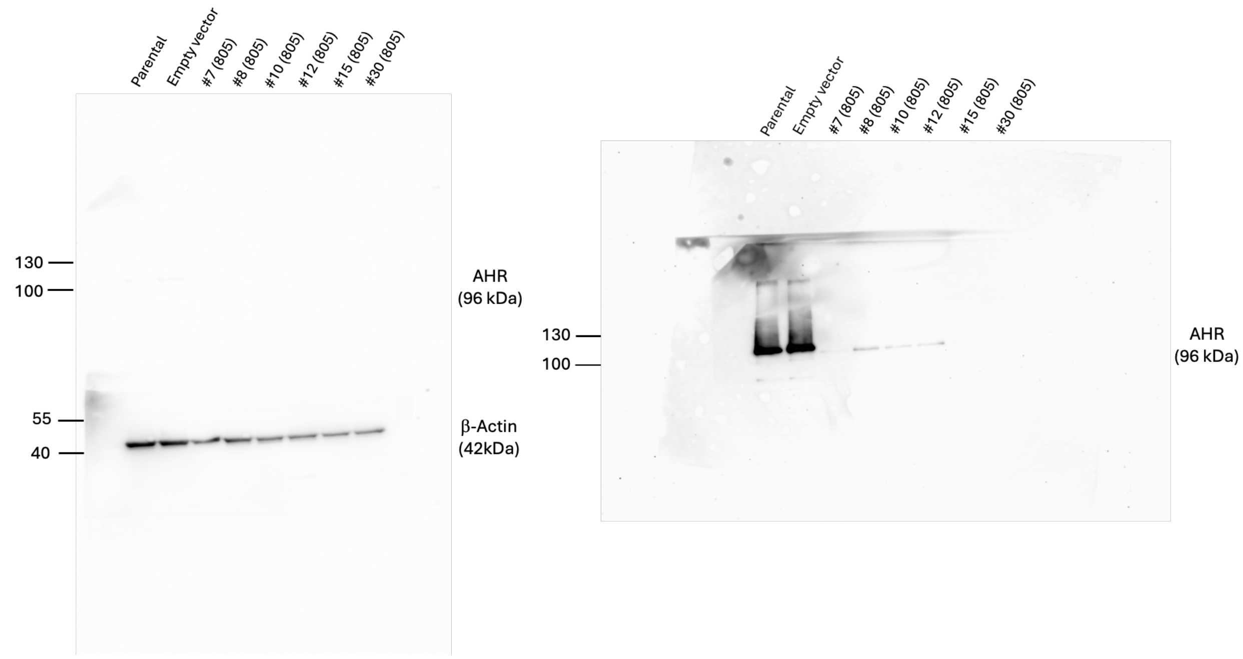

Data S1: An original uncropped image of Figure S4C.
